# Aerosolization flux, bio-products, and dispersal capacities in the freshwater microalga *Limnomonas gaiensis* (Chlorophyceae)

**DOI:** 10.1038/s42003-023-05183-5

**Published:** 2023-08-03

**Authors:** Sylvie V. M. Tesson, Marta Barbato, Bernadette Rosati

**Affiliations:** 1 Aarhus Institute of Advanced Studies, Aarhus University, Aarhus, Denmark; 2 Department of Biology, Aarhus University, Aarhus, Denmark; 3 Department of Chemistry, Aarhus University, Aarhus, Denmark

**Keywords:** Ecology, Microbiology

## Abstract

Little is known on the spreading capacities of *Limnomonas gaiensis* across freshwater lakes in Northern Europe. In this study, we show that the species could successfully be aerosolized from water sources by bubble bursting (2-40 particles.cm^−3^), irrespectively of its density in the water source or of the jet velocity used to simulate wave breaking. The species viability was impacted by both water turbulences and aerosolization. The survival rate of emitted cells was low, strain-specific, and differently impacted by bubble busting processes. The entity “microalga and bionts” could produce ethanol, and actively nucleate ice (principally ≤−18 °C) mediated soluble ice nucleation active proteins, thereby potentially impacting smog and cloud formation. Moreover, smallest strains could better cope with applied stressors. Survival to short-term exposure to temperatures down to −21 °C and freezing events further suggest that *L. gaiensis* could be air dispersed and contribute to their deposition.

## Abstract (web summary)

*Limnomonas gaiensis* can be aerosolized by bubble bursting at a low survival rate. Its tolerance to subzero temperatures and the microalga-biont ability to produce ethanol and nucleate ice suggest they could disperse and be of atmospheric importance.

## Introduction

The microalga *Limnomonas gaiensis* inhabits freshwater lakes in Northern Europe. This member of the *Chlamydomonas* phylogroup has recently been morphologically and genetically described^1^. The species has been isolated from unconnected water systems in Northern Europe^1^ and presents key features for organismal dispersal and local adaptation characterized by a potential to acclimate to a wide range of pH^2^. However, its spreading capacity is not known.

Airborne *Chlamydomonas* species have been reported from a wide range of geographical locations^3^, with successful dispersal^4–6^ including in the snow species *C. nivalis*^7^. Because of the distance and absence of water connectivity between lakes where *L. gaiensis* occurs, we hypothesized that air dispersal may play a role in its spreading.

Aquatic microalgae are aerosolized by water surface abrasion^3^, via wind-friction and breaking wave crests generating spume drops^8^, or by bubble bursting producing film or jet droplets. Microalgal aerosolization has been reported over land and ocean, with variations according to location, wind conditions^3,9^, organismal density in the water source, and growing conditions^10,11^. To date, less than a hand-full emission fluxes are available^4,12–14^ reaching up to 3 × 10^3^ cells.m^−3^ in microalgae and 4 × 10^5^ cells.m^−3^ in picomicroalgae (0.2–2 µm). Moreover, processes governing microalgal aerosolization are still poorly characterized.

Microalgal aerosolization has retained attention because of their capacities at interacting with the atmosphere, adapting morphologically to survive atmospheric conditions^15^, dispersing to new environments, and being a source of sanitary risks for the environment and the society^3,9,16^. Proliferation of airborne microalgae, such as *Chlamydomonas* spp.^16^, can lead to severe environmental and sanitary issues both indoors^16,17^ and outdoors^3,9,18^. Moreover, some can proliferate in lakes contaminated by toxic cyanobacteria^6^, a similarity with *L. gaiensis*^1^.

Microalgae can produce volatile organic compounds (VOCs) that are important for atmospheric chemistry^19–22^. They can also actively nucleate ice from below −6 °C mediated the production of ice nucleation active (INA) compounds^23^ and from below −23 °C through the production of INA exudates^24^. More specifically, certain *Chlamydomonas* sp. can actively nucleate ice^25^ at temperature comprised between −8 and −17 °C. Therefore, produced microalgal VOCs and INA molecules can have potential impact on atmospheric processes such as clouds formation, and their own deposition.

Aerosolized microalgae are not expected to stay airborne for prolonged periods of time due to their typically large sizes^3^. For this reason, their impact on climate and spreading mediated air transportation has been thought to be negligible. Surprisingly, some microalgae have been reported far away from their potential sources, even in remote locations such as Antarctica^3,26–28^. Additionally, using back trajectory analysis, studies have shown that long and viable transportation of microalgae was feasible^15,29–31^. The long air transportation gives rise to several opportunities of interactions with solar radiation and increases their potential to act as so-called giant cloud condensation nuclei (CCN) forming the seed for liquid cloud droplets to form. The role of airborne microalgae as giant CCN has previously been suggested^28^ but is so far not well understood.

This study aims to infer if *L. gaiensis* can be emitted into the atmosphere via bubble busting processes and cope with stresses associated with the emission, and to identify biotic compounds that could influence atmospheric processes. We investigated its (i) aerosolization capacity (i.e., relative magnitudes of aerosolization fluxes) for different types of jets, (ii) effect on atmospheric chemistry (i.e., production of VOCs and of ice nucleation active compounds from −4 down to −21 °C), and (iii) viable capacities after aerosolization and freezing events. Results from our study may provide novel insights in microalgae dispersal and their role as primary biological aerosol particles (PBAPs) in the atmosphere.

## Results

### Pilot study

In a pilot study (Experiment (Exp)1–2, Table 1), we investigated the impact of bubble bursting (Single Jet (SJ) versus Multiple Jets (MJ)), at different water flow intensities (SJ7, SJ9, MJ5, MJ3), on the emission of VR66-07 and R86-47. In all scenarios, results showed that microalgae were successfully aerosolized by bubble bursting.

**Table 1:** Aerosolization settings.

Experiment	Strain	Abundance (cells)	Concentration (cells.L^−1^)	Flow	Date	Collector	Emitted cells captured (cells)
Exp1	VR66-07	36.1 × 10^6^	3.56 × 10^6^	SJ7, SJ9, MJ5, MJ3	2021-11-16	Filters	SJ: 2109 ± 788 MJ: 2133 ± 377
Exp2	R86-47	26.0 × 10^6^	2.57 × 10^6^	SJ7, SJ9, MJ5, MJ3	2021-11-17	Filters	SJ: 2933 ± 943 MJ: 3167 ± 2027
Exp3	R86-47	19.0 × 10^6^	1.87 × 10^6^	SJ9, MJ3	2021-12-14	Filters	SJ: 3600 MJ: 4600
Exp4	VR66-07	19.2 × 10^6^	1.89 × 10^6^	SJ9, MJ3	2021-12-15	Filters	SJ: 1552 MJ: 2400
Exp5	VR66-07	17.5 × 10^11^	1.67 × 10^11^	MJ3, SJ9	2022-07-11	Impingers	MJ: 37875000 ± 10980096 SJ: 39375000 ± 12023415
Exp6	R86-47	29.2 × 10^8^	2.77 × 10^8^	MJ3, SJ9	2022-07-12	Impingers	MJ: 26667 ± 7201 SJ: 42500 ± 6161
Exp7	R86-47	40.0 × 10^7^	3.72 × 10^7^	SJ9, MJ3	2022-10-12	Impingers	SJ: 217222 ± 21104 MJ: 251667 ± 20207

Consecutive experiment (Exp) number, the corresponding investigated strain of *Limnomonas gaiensis*, the total microalgal abundance and concentration in homogenized tank at the beginning of the experiment, the water treatment used (Single-Jet (SJ) at intensities 7 or 9, Multiple Jets (MJ) at intensities 3 or 5), the experimental date, the devices use to collect airborne particles, and the number of emitted cells captured on device. *NA* not available.

Direct measurement of the emitted microalgae (EM) ensemble (i.e., cells, cell fragments and other microalgal material) was estimated using the Optical Particle Spectrometer (OPS) (Supplementary Figs. 1 and 2). Exp1 data was excluded from the emission flux analysis due to a malfunctioning in the OPS pump. Exp2 showed a constant emission of aerosolized particles over time in SJ and MJ. The flow rates SJ9 and MJ3 yielded the largest concentrations of emitted particles (Supplementary Fig. 2). In the light of these results, we applied SJ9 and MJ3 to the successive experiments.

The abundance of EM, captured onto filters over Exp1-2, was assessed using Lugol fixed samples. In all samples, numerous cell fragments were visible. Unexpectedly, the number of intact EM was higher after SJ (2467 cells ± 336) than after MJ (1800 cells ± 255) (one-way ANOVA *F*_(1,6)_ = 10.0, *p* = 0.020), in both strains (*F*_(1,6)_ = 0.67, *p* = 0.45). We suspected that the filtration pressure may have damaged EM integrity and affected the results. To avoid bias, an alternative technique in liquid phase was used in Exp5–7 to capture EM.

Indirect estimation of the number of EM was calculated from Lugol fixed samples collected from the water tank (Supplementary Figs. 1–3), showing a significant decrease in the abundance of aquatic microalgae (−73.8% ± 0.4 cells on average; Kruskal–Wallis *X*^2^_(4)_ = 21.94, *p* < 0.001, Dunn test *p* < 0.001), in both strains (*X*^2^_(1)_ = 0.004, *p* = 0.95). Despite the percentage of EM after MJ (51.2% ± 19.9) was higher than after SJ (41.7% ± 22.9), there was no significant difference between the two treatments (Fisher Exact probability test, *p* = 0.26), possibly due to the high standard deviation retrieved from the counts. Discrepancies regarding Lugol counts over time are known^32^, and therefore a more systematic approach was used to estimate abundances, e.g., counting replicates of a sample phase within a day and applying the same dilution factors for all replicates.

### Aerosolization

**Fig. 1:**
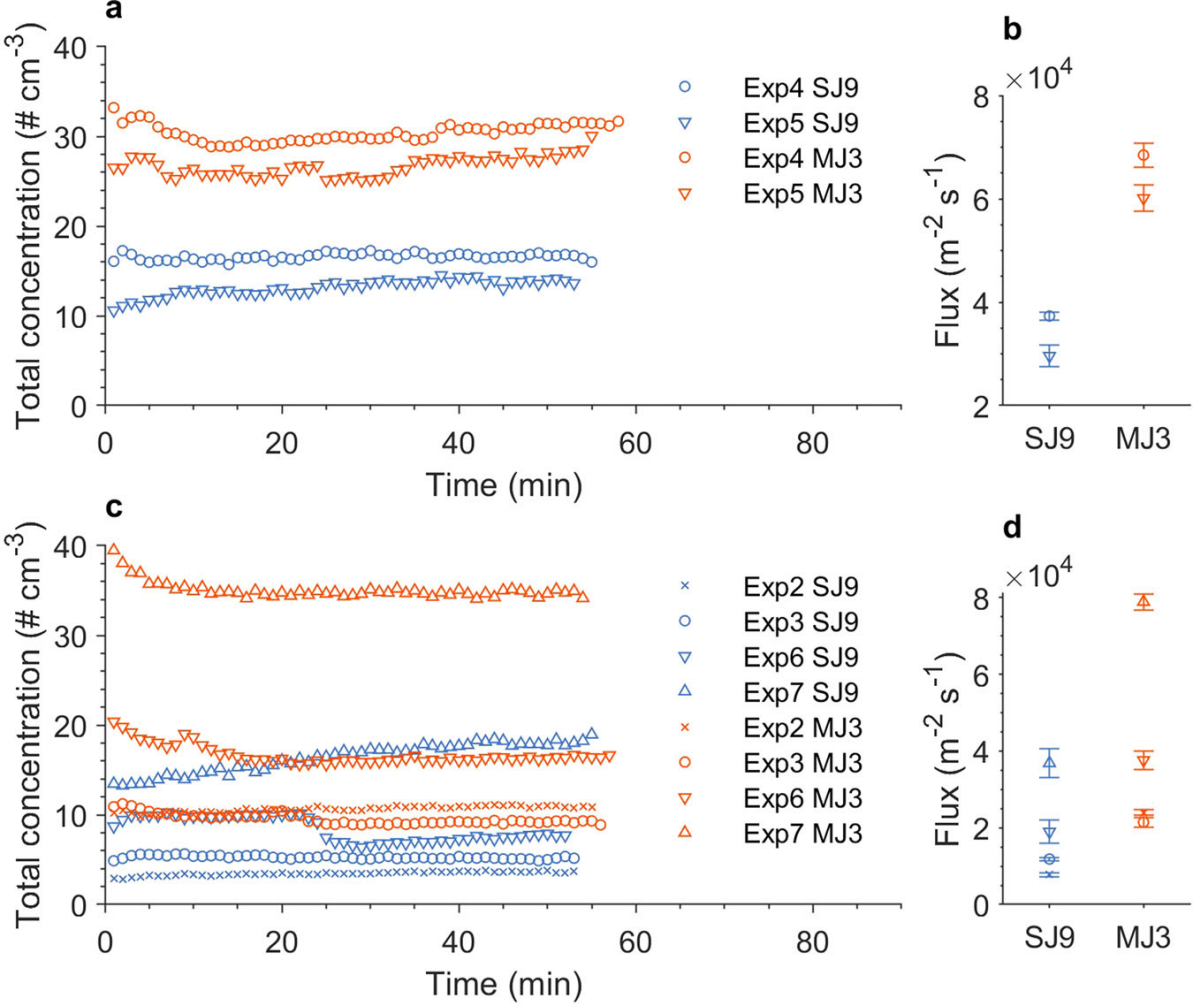
Total number concentration and flux of aerosolized microalgae ensemble. The time series illustrating the total concentration of aerosolized particles (**a**, **c**) under SJ9 (blue) and MJ3 (orange) treatments in two strains of *Limnomonas gaiensis*, i.e., VR66-07 (**a**, **b**) and R86-47 (**c**, **d**), in Experiment 2 (crosses), Experiments 3–4 (circles), Experiments 5–6 (upside-down triangles), and Experiment 7 (upside-up triangles). Additionally, the mean flux is illustrated for each of the two strains (b,d), for both treatments, together with one standard deviation denoting the variability over time (*n* = 52–58).

The OPS data confirmed that both strains were emitted under SJ9 and MJ3 (Table 1 and Fig. 1). The total concentration of EM ensemble ranged from 2 to 40 particles.cm^−3^ and was relatively constant in all treatments and strains (Fig. 1). The emission fluxes, ranging 0.8–7.9 × 10^4^ m^−2^.s^−1^, were comparable between the two strains (one-way ANOVA *F*_(1,10)_ = 2.16, *p* = 0.17) and were systematically, but only marginally significantly smaller under SJ9 (2.4 × 10^4^ m^−2^.s^−1^ ± 1.3 × 10^4^) than MJ3 (4.8 × 10^4^ m^−2^.s^−1^ ± 2.4 × 10^4^) (Fig. 1, Kruskal–Wallis *X*^2^_(1)_ = 3.69, *p* = 0.055). Moreover, there was no clear relation between the emission flux and microalgal abundance in the water tank (Supplementary Fig. 4).

The mean number of EM captured by filters (solid and dry phase, Exp1–4) was 2586 cells (± 876), and numerous broken cells and debris were visible. Captured cell number did not differ significantly between treatments (Kruskal-Wallis *X*^2^_(1)_ = 0.53, *p* = 0.47), nor strains (one-way ANOVA *F*_(1,10)_ = 1.04, *p* = 0.33). The number of EM captured by impingers (liquid phase, Exp5–7) was at least 10 times higher than using filters (Table 1), with few to no broken cells visible. However, the proportion of cells captured by the two devices were fairly similar as the microalgal abundance in the emission source was >10 times higher in Exp5–7 than in Exp1–4 (Table 1). Captured cell numbers by impingers did not differ significantly between treatments (Kruskal–Wallis *X*^2^_(1)_ = 0.017, *p* = 0.90), but between strains (Kruskal–Wallis *X*^2^_(1)_ = 14.63, *p* < 0.001).

Indirect measurement confirmed the aerosolization of both strains from the water source with 32.8% (± 24.6) of EM. Despite a slightly higher EM rate under SJ (41.1% ± 25.1) than MJ (24.5% ± 23.3), this emission was not significantly affected by the choice of treatment (two-way ANOVA *F*_(1,8)_ = 1.21, *p* = 0.30) nor by strains (*F*_(1,8)_ = 0.58, *p* = 0.47), or by strains and treatment (*F*_(1,8)_ = 0.062, *p* = 0.81). The fluctuation in microalgal abundance in the water tank (Table 2) was not significantly affected by treatments (Kruskal–Wallis *X*^2^_(1)_ = 2.37, *p* = 0.12) or strains (*X*^2^_(1)_ = 0.009, *p* = 0.93). Notable fluctuations in microalgal abundances in the water tank were observed between experiments (Kruskal-Wallis *X*^2^_(6)_ = 32.59, p < 0.001) as a result of different microalgal load in the water tank (*X*^2^_(4)_ = 38.99, *p* < 0.001), in particular between Exp1–4 and Exp5–7 (Dunn Test *p* < 0.05). When 10^7^ cells were loaded in the tank (Exp1–4), the microalgal abundances in the water tank remained higher under SJ than MJ (Kruskal–Wallis *X*^2^_(1)_ = 12.81, *p* < 0.001), resulting in lower emission fluxes under SJ, as observed in the OPS data (Fig. 1). However, when higher microalgal abundances were loaded (Exp5–7), there was no significant difference between treatment (Kruskal–Wallis *X*^2^_(1)_ = 0.11, *p* = 0.74), despite systematically higher fluxes were recorded by the OPS under MJ (Fig. 1).

**Table 2:** Abundance of cells of *Limnomonas gaiensis* in water tank after single or multiple jet treatment.

Experiment	Strain	Abundance SJ (10^6^ cells)	Abundance MJ (10^6^ cells)
Exp1	VR66-07	15.21 ± 0.00	9.56 ± 0.97
Exp2	R86-47	19.40 ± 11.37	6.74 ± 2.33
Exp3	R86-47	9.49 ± 0.68	8.12 ± 0.68
Exp4	VR66-07	11.08 ± 3.21	8.81 ± 1.17
Exp5	VR66-07	1672160.49 ± 22158.87	1674842.40 ± 54317.17
Exp6	R86-47	817.91 ± 169.47	2762.38 ± 100.93
Exp7	R86-47	586.31 ± 81.72	1067.50 ± 546.48
Mean	VR66-07	557395.59 ± 965414.72	558286.92 ± 966965.41
Mean	R86-47	358.28 ± 408.15	961.18 ± 1300.63

The seven experiments (Exp) performed. Strains: VR66-07 and R86-47. Treatment: single jet (SJ) or multiple jets (MJ). No significant difference in cell abundances between strains (*X*^2^_(1)_ = 0.54, *p* = 0.46) nor treatments (*X*^2^_(1)_ = 1.54, *p* = 0.21) nor strains and treatments (Dunn test *p* > 0.47). Pairwise significant differences between experiments, between Exp1–4 and Exp5–7 (Dunn test *p* < 0.03, marginally significant for Exp1 vs Exp7 *p* = 0.06).

### Biovolume

**Fig. 2:**
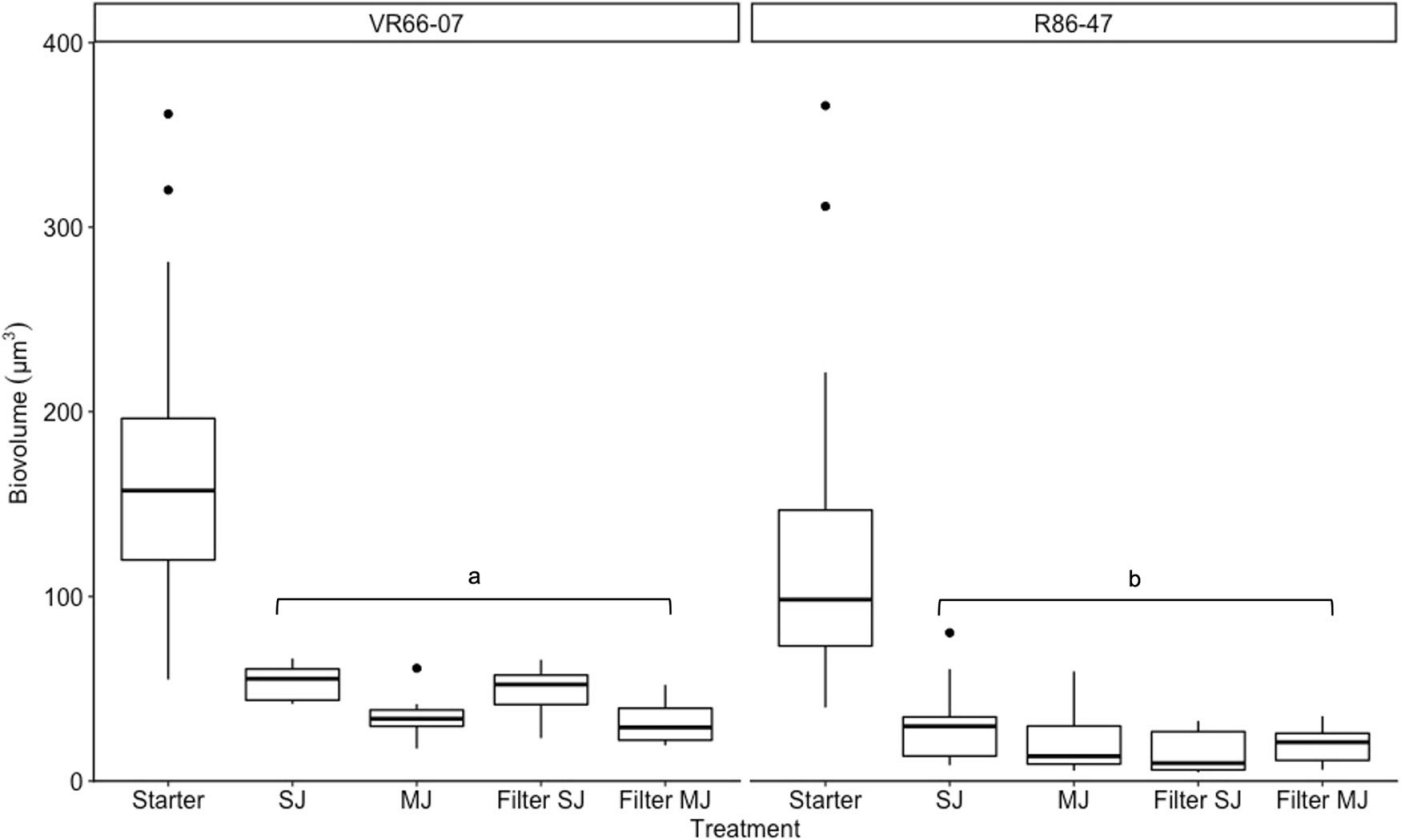
Biovolume distribution of *Limnomonas gaiensis* collected in water tank and captured on filter device after SJ and MJ treatments. Box plot of the biovolume values measured in two strains of *L. gaiensis*: VR66-07 (Experiment 4) and R86-47 (average of Experiments 2–3). Number of observations are comprised between 6 and 50 per strain and treatment. Significant differences between strains through the experiment was observed (a vs b, *p* = 0.036).

Strain biovolume was estimated from the water source and filters (Exp2–4, Fig. 2) to investigate if size selection occurred during emission. Both strains showed the same trend in all experiments (two-way ANOVA strains: *F*_(1,6)_ = 2.60, *p* = 0.16, experiments: *F*_(1,6)_ = 0.036, *p* = 0.86). A significant decrease in biovolume was observed between the starter (original culture) and the treatments (*F*_(4,6)_ = 11.91, *p* = 0.0051). The cell biovolume was not affected by SJ or MJ in organisms collected on filters (TukeyHSD *p* = 0.99) nor in the water tank (TukeyHSD *p* = 0.99). But difference in biovolume was observed between strains under treatment (one-way ANOVA *F*_(1,8)_ = 6.374, *p* = 0.036), with an average smaller biovolume in R86-47 (24.0 µm^3^ ± 11.6) than in VR66-07 (42.3 µm^3^ ± 10.5).

### Revival capacities after aerosolization

To understand the constrains of treatment and recapture on *L. gaiensis* potential for air dispersal, the revival capacity of EM was investigated. None of the inoculates collected from filters were able to revive or show vital signs (growth or movement) under condition favoring growth over a two-month period of incubation. Instead, numerous fragmented cells and debris were visible. Results suggested that only a small proportion of EM may be viable, or, that the EM were negatively impacted by filtration pressure.

Inoculates collected from impingers showed 10.4% (± 4.8; 57/548 inoculates) survival success, suggesting that capture in liquid phase may break less cells and that a small portion of microalgal strains could be airborne dispersed. The two strains reacted differently to bubble bursting treatments, with a lower survival rate in VR66-07 (4.9%; 9/184 inoculates; Exp5) than in R86-47 (13.2% ± 1.0, Exp6-7; 48/364 inoculates) (Z-test *X*^2^_(1)Exp5-Exp6_ = 5.78, *p* = 0.016; *X*^2^_(1)Exp5-Exp7_ = 7.67, *p* < 0.01). More specifically, significant differences were observed for MJ (VR66-07: 0% (0/92 inoculates) vs R86-47: 14.8% ± 0.5 (27/182 inoculates); Fisher’s Exact Test for Count Data *p*_MJ_ < 0.0001), but not SJ (VR66-07: 9.8% (9/92 inoculates) vs R86-47: 11.6% ± 2.5 (21/182 inoculates), strains: 11.0% ± 2.1 (30/274 inoculates), Fisher’s Exact Test for Count Data *p*_SJ_ = 0.81). Results were reproducible between experiments (*X*^2^_(1)Exp6-Exp7_ = 0.06, *p* = 0.81) and treatments (*Z*-test *X*^2^_(1)MJ_ < 0.001, *p* = 1.0; *X*^2^_(1)SJ_ = 0.27, *p* = 0.61).

### Cell vitality

Organism survival capacity was also assessed using live/dead stains in samples collected from the water tank and after emission. The Neutral Red vital stain was used to infer the proportion of intact organisms. It efficiently stains several microalgal taxa^23,33^ but did not properly stain *L. gaiensis* as the orange-red signal of the dye was masked by the microalgal cup-shaped morphology of the chloroplast, hiding signals from underneath vacuoles and cytoplasm.

**Fig. 3:**
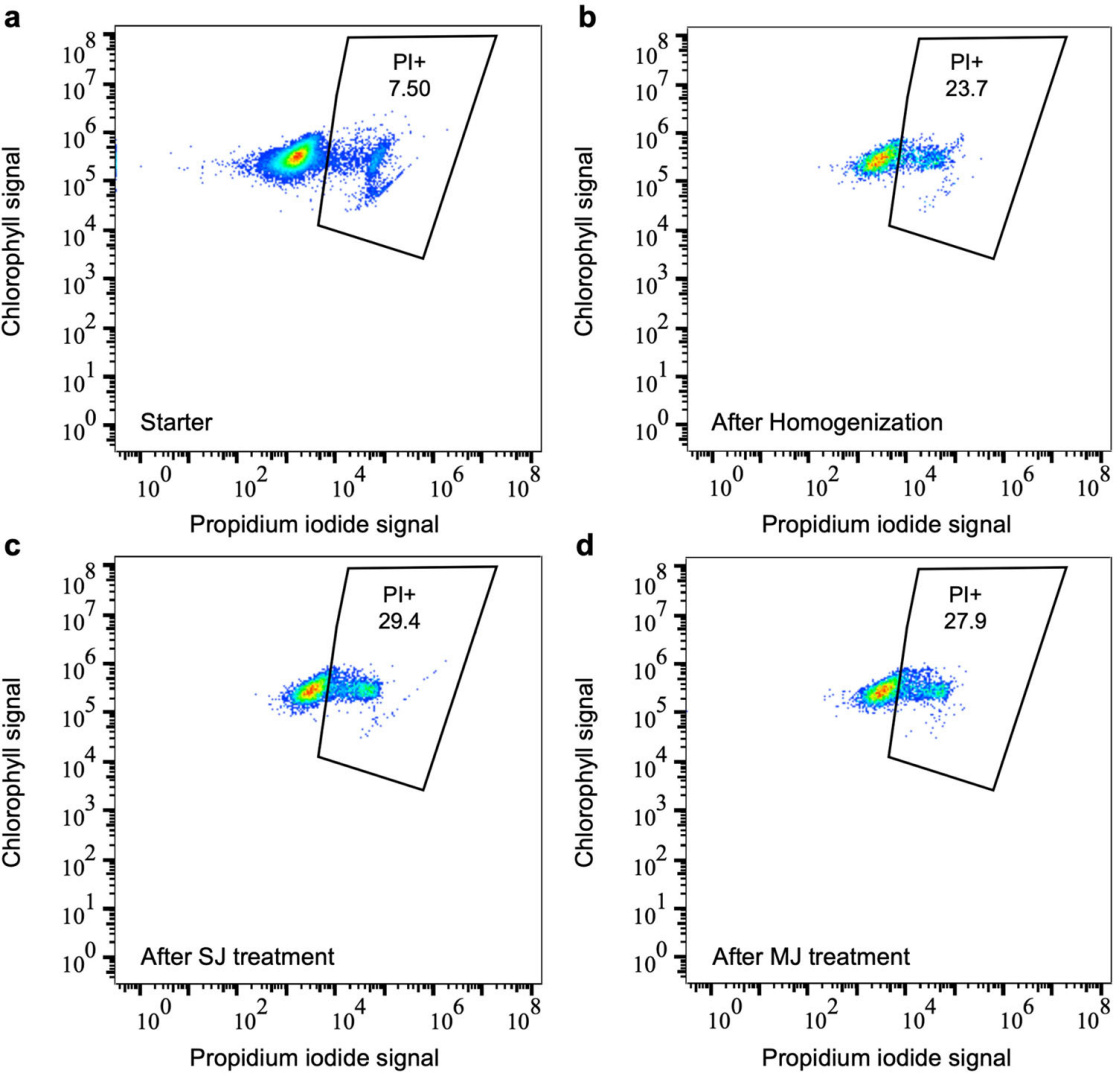
Distribution of chlorophyll-containing cells and dead cells in Experiment 7. Dead cells stained by propidium iodide (PI+; cluster on the right) with an indication of the percentage of dead cells among chlorophyll-containing cells (*n*_total_). Treatments include the initial culture (**a**, *n*_total_ = 0.9 × 10^9^ cells), after water homogenization (**b**, *n*_total_ = 1.6 × 10^9^ cells), after SJ9 treatment (**c**, *n*_total_ = 2.4 × 10^9^ cells), and after MJ3 treatment (**d**, *n*_total_ = 2.4 × 10^9^ cells).

Propidium iodine was used to assess the fraction of dead/damaged organisms (Exp7, Supplementary Table 1). The percentage of dead cells was low in the starter (7.0% ± 0.5). It significantly increased to 25.5% (± 3.5) after water homogenization (Fisher’s Exact Test for Count Data *p* < 0.001) and remained constant across treatments in the aquatic population (SJ: 28.4% ± 2.3, MJ: 27.9% ± 1.6; Fisher’s Exact Test for Count Data *p*_Homogenization-Treatment_ = 0.87 and *p*_SJ-MJ_ = 1.0). The dynamic of dead cell occurrence investigated using flow cytometry (Fig. 3) confirmed a net diminution of the intact cell population (outside the gate) towards dead cells (inside the gate), principally after water homogenization (Fig. 3a, b), indicating that water turbulence, regardless of SJ or MJ (Fig. 3c, d), had a negative impact on cell survival. The percentage of dead cells was very high in the emitted fraction (97.1% ± 4.2), corroborating with the low revival rate retrieved from the inoculates. There was a marginally significantly higher proportion of dead cells under SJ than MJ (Fisher’s Exact Test for Count Data *p* = 0.06). However, this tendency is to take with caution because the total number of cells detected by the flow cytometer in the emitted fraction was quite low and in the range of the detection threshold.

### VOCs

**Fig. 4:**
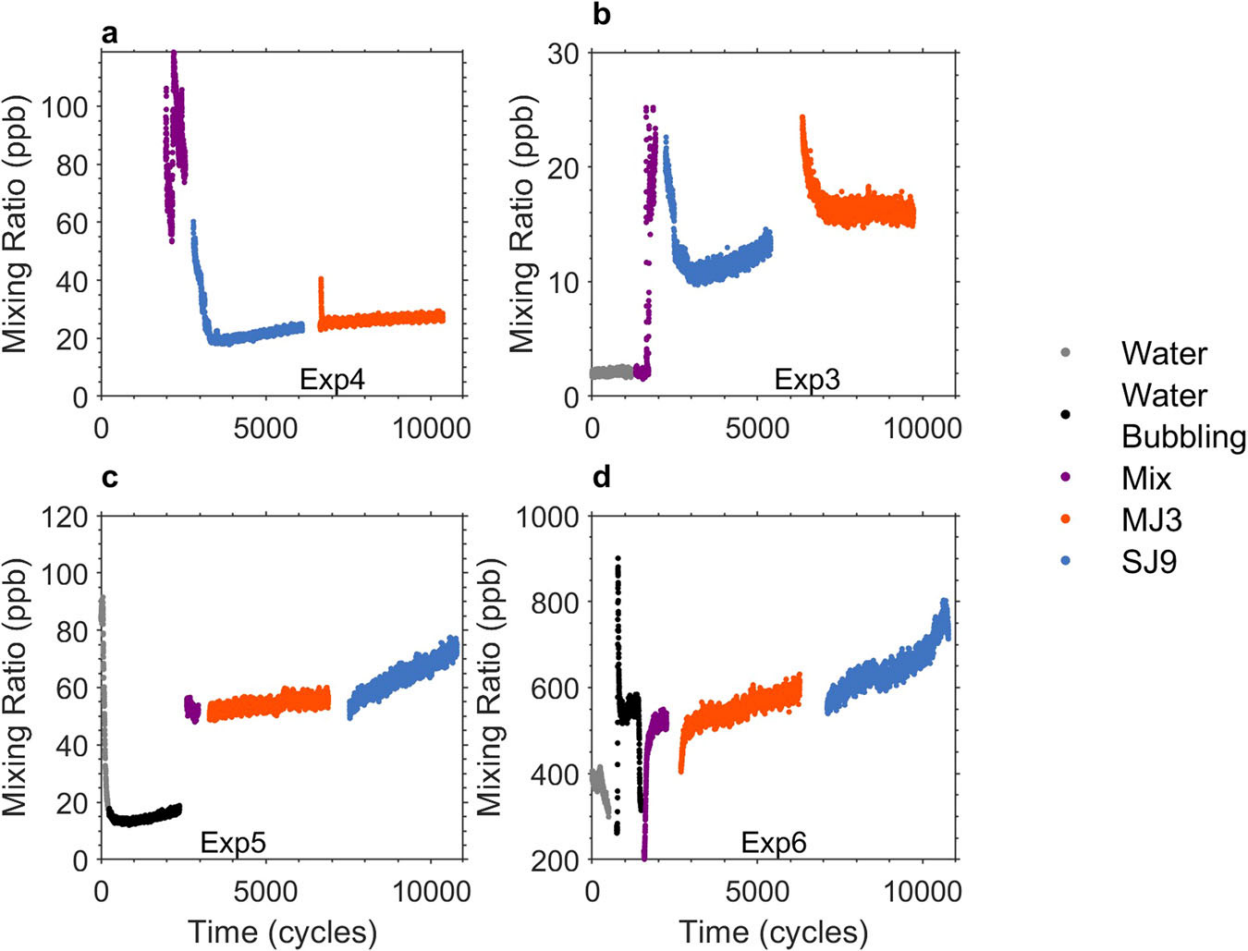
Timeseries of volatile organic compound emissions of aerosolized microalgae ensemble. Panels **a** and **c** illustrate results for two experiments with the *Limnomonas gaiensis* strain VR66-07 over Experiments 4 and 5, respectively, and panels **b** and **d** show the results with strain R86-47 over Experiments 3 and 6, respectively. Only the VOC m/z 47.05 was detected and corresponds to the protonated signal for ethanol. Five different phases of the experiments are highlighted in different colors, e.g., emission from MilliQ water (gray dots, *n* = 212–1200), emissions from bubbling MilliQ water (black dots, *n* = 757–2134), emissions during first mixing (homogenization, purple dots, *n* = 351–685), emissions from MJ3 (orange dots, *n* = 3360–3720) and SJ9 (blue dots, *n* = 3180–3670) treatment.

VOCs production is of relevance for their potential effect on atmospheric chemistry (e.g., climate, cloud formation, see Discussion). Gaseous emissions of *L. gaiensis* natural entity (i.e., microalga and bionts, see Methods) were investigated over the experimental phases. VOCs in the m/z range 21–200 were explored and the emission of m/z 47.05 (ethanol) under stress was found. Despite ethanol is used for cleaning purposes and its mixing ratio in the laboratory air varied substantially between experiments, *L. gaiensis* entity was able to produce ethanol under stress (Fig. 4).

Ethanol emissions were low when the tank was filled only with water (water controls) and higher when the *L. gaiensis* entity was present (treatments, Fig. 4). A strong initial decrease in ethanol concentration was recorded over time when only water was in the tank (water condition, Fig. 4 Exp5-6), reflecting the replacement of laboratory air in the tank headspace by filtered air. The ethanol signal strongly increased when organisms were homogenized (mix condition, Fig. 4). A steady increase in ethanol emission was observed with SJ9, characterized by a slope of 0.006 ppb.s^−1^ (Exp3) and 0.040 ppb.s^−1^ (Exp6) in R86-47 and of 0.002 ppb.s^−1^ (Exp4) and 0.006 ppb.s^−1^ (Exp5) in VR66-07. It also increased during MJ3 in R86-47 with a slope of 0.001 ppb.s^−1^ (Exp3) and 0.03 ppb.s^−1^ (Exp6) but remained constant in VR66-07.

### Frozen fraction and IN activity

**Fig. 5:**
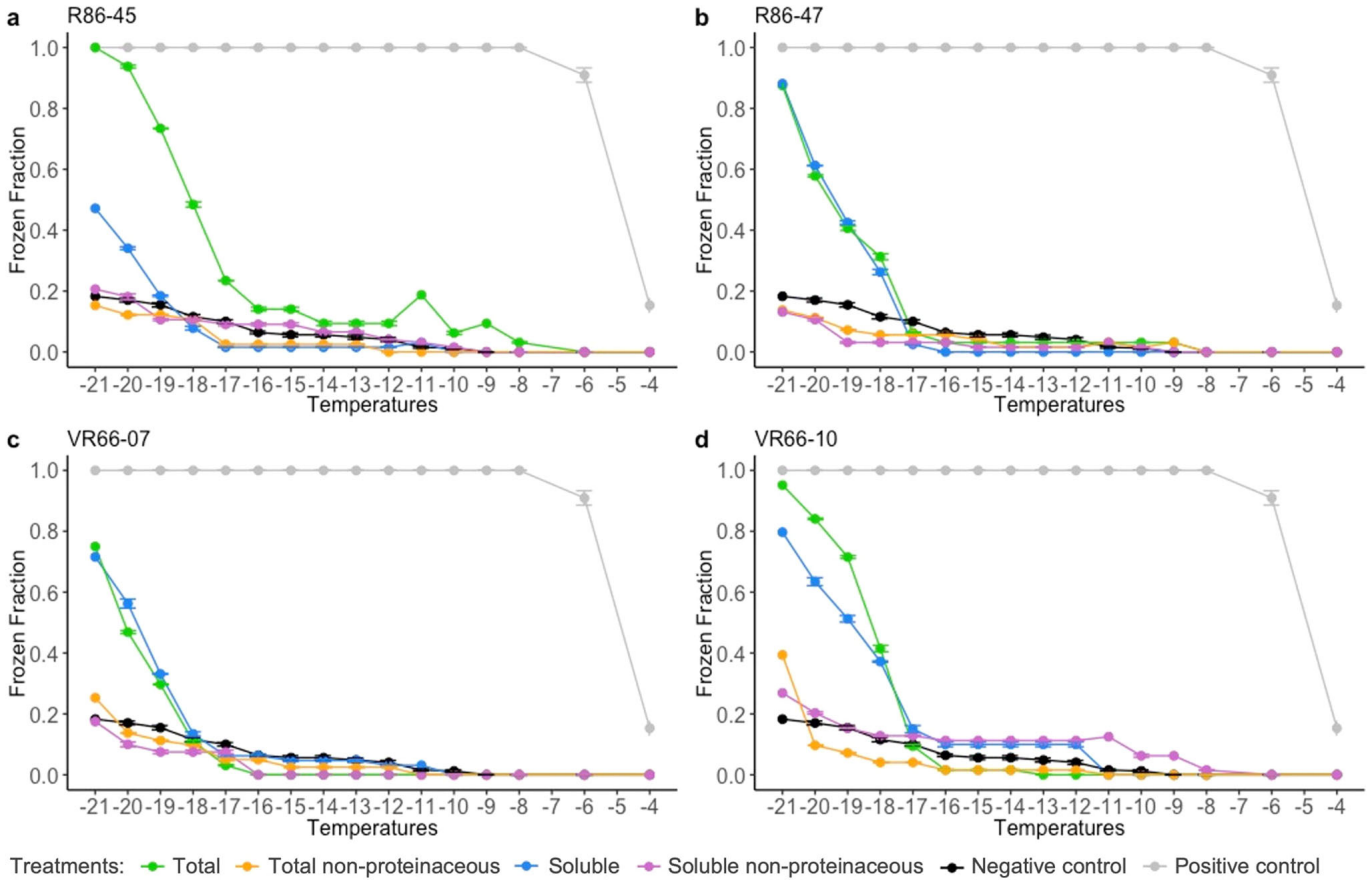
Freezing profiles in four strains of *Limnomonas gaiensis* to investigate the nature of ice nucleation active compounds and the temperature spectra at which this reaction occurs. The four investigated strains of *Limnomonas gaiensis*, R86-45 (**a**), R86-47 (**b**), VR66-07 (**c**), and VR66-10 (**d**) exposed to a decreasing gradient of −1 °C from −4 to −21 °C. The total fraction is indicated in green. Treatments included either protein denaturation by heat (non-proteinaceous, orange), molecule size selection by filtration on 0.22 µm pore membrane (soluble, blue), or a combination of both treatments (soluble non-proteinaceous, purple). The number of replicates was of 32 droplets of 20 µL of either microalgae (strain), MWC (negative control, black) or *Pseudomonas syringae* active >-4 °C (positive control, gray). The error bars show the 95% confidence interval. Each data point is the synthesis of a total of 52 to 64 replicates per strain and treatment, and of 116 replicates per control.

**Fig. 6:**
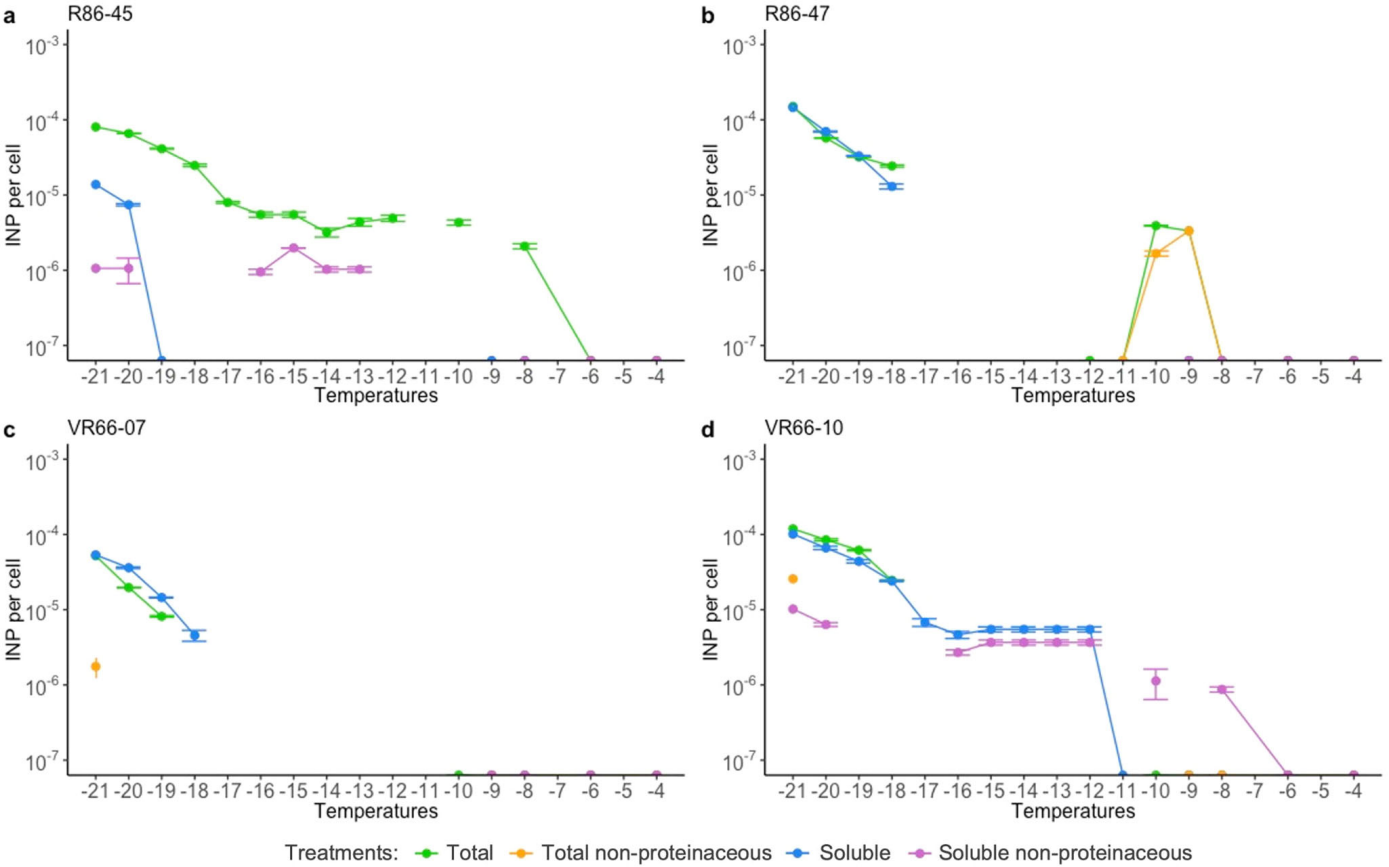
Concentration of ice nucleating particles produced by *Limnomonas gaiensis* strains. The four investigated strains of *Limnomonas gaiensis*, R86-45 (**a**), R86-47 (**b**), VR66-07 (**c**), and VR66−10 (**d**) exposed to a decreasing gradient of −1 °C from −4 to −21 °C. Data are normalized to the negative control. The total fraction is indicated in green. Treatments included either protein denaturation by heat (non-proteinaceous, orange), molecule size selection by filtration on 0.22 µm pore membrane (soluble, blue), or a combination of both treatments (soluble non-proteinaceous, purple). The error bars show the 95% confidence interval for 52–64 replicates per strain and treatment.

The capacity of *L. gaiensis* entity to actively nucleate ice was investigated using droplet-freezing assays in four strains^1^. Freezing events and the number of ice nucleating particles (INP) (total fraction, Figs. 5, 6) were estimated when exposed to a gradient spanning −4 to −21 °C. Strains originating from Lake Ryssbysjön started freezing at −8 °C, i.e., R86-45 at >−8 °C with 2.1 × 10^−6^ INP.cell^−1^ and R86-47 at <−8 °C with 3.3 × 10^−6^ INP.cell^−1^. Strains from Lake Västra Ringsjön were active at lower subzero temperatures, i.e., VR66-10 at <−17 °C with 2.5 × 10^−5^ INP.cell^−1^ and VR66-07 at <−18 °C with 8.2 × 10^−6^ INP.cell^−1^. In R86-47 the IN activity remained low, between −8 and −17 °C ( ≤ 3.9 × 10^−6^ INP.cell^−1^), sometimes below the detection limit (<−12 °C) and started to increase again at <−17 °C ( ≤ 1.5 × 10^−4^ INP.cell^−1^). In all strains, half of the replicates were frozen (frozen fraction (FF) of 0.5) from −18 down to −21 °C (Fig. 5). At −21 °C, all replicates were frozen (FF = 1) in R86-45 (Fig. 5a). In the three other strains (Fig. 5b–d), FF reached 0.95 in VR66-10, 0.88 in R86-47 and 0.75 in VR66-07. At −21 °C the number of INP was 1.01 × 10^−4^ (± 0.4 × 10^−4^) INP.cell^−1^ on average, reaching 8.2 × 10^−5^ INP.cell^−1^ in R86-45, 1.5 × 10^−4^ INP.cell^−1^ in R86-47, 5.2 × 10^−5^ INP.cell^−1^ in VR66-07, and 1.2 × 10^−4^ INP.cell^−1^ in VR66-10 (Fig. 6). Results indicated that *L. gaiensis* entity could be IN active at rather low temperatures, almost negligeable compared to known INA PBAPs (e.g., *P. syringae*, our positive control, ≤−6 °C) and abiotic particles (≤−12 °C).

The nature of the component responsible for IN activity was investigated using heat and filtration treatments (Fig. 5). There was a significant effect of treatments on strain activity (Kruskal–Wallis *X*^2^_(15)_ = 26.0, *p* = 0.04, Fig. 6), and in particular an effect of heat (Dunn Test *p* = 0.002), suggesting that the INA compounds were proteinaceous. Filtration treatment was performed to decipher if the proteinaceous INA compounds were soluble (size <0.22 µm). Strains R86-45 was significantly affected by the filtration treatment (Figs. 5a and 6a, Dunn Test *p* = 0.03), indicating that its INA compounds were not soluble. However, in the three other strains, the effect of filtration was not significant (Dunn Test *p* = 0.24, *p* = 0.63, *p* = 0.61, respectively), suggesting soluble INA compounds (Figs. 5b–d and 6b–d). When both the total and the soluble fractions were heated, there was a significant drop in the IN activity, below the detection limits (Figs. 5–6), confirming the proteinaceous origin of the INA compounds. In summary, results indicated that the IN activity was triggered by INA proteins that were either soluble (R86-47, VR66-07, VR66-10) or associated to the organisms (R86-45).

Additionally, the peak of IN activities in R86-47 between −8 and −11 °C and in VR66-10 from −12 down to −17 °C were investigated (Fig. 6b, d). R86-47 was sensitive to filtration (Dunn Test *p* = 0.09) but not to heat treatment (Dunn Test *p* = 0.41), indicating that its INA compounds were non-proteinaceous possibly associated to the organism (e.g., exudates). VR66-10 showed IN activity only in the soluble fraction over this temperature interval, but not in the total fraction. Moreover, a significant reduction of activity between the soluble and the soluble heated fractions indicated that the soluble INA compounds were proteinaceous (Kruskal-Wallis *X*^2^_(1)_ = 8.25, *p* = 0.004, Dunn Test *p* = 0.004). Results suggested that the filtration pressure in VR66-10 may have affected the integrity of some cells and released soluble INA proteins in the filtrated fractions.

### Revival capacities after cold exposure and freezing events

The revival capacity of *L. gaiensis* strains was investigated after exposure to thermal stress gradually decreasing down to −21 °C and an incubation period of up to three weeks at conditions favoring growth. The four strains were able to cope with up to 8 h exposure to these subzero temperatures. About half of the replicates revived (183/384 replicates). R86-47 showed the highest survival rate (59–100%) and R86-45 the lowest (0–63%). There was a general decrease in the revival percentage with the increment in cell abundance (Supplementary Fig. 5). The highest percentage of revival was observed in the “youngest tested culture”, having the lowest abundance (Supplementary Table 2) and undergoing exponential growth phase (color-based observation). Interestingly, a third of the revival (58/183 replicates) occurred in wells that experienced freezing, suggesting that *L. gaiensis* strains could survive freezing events.

## Discussion

This study revealed that *L. gaiensis* could be aerosolized from freshwater sources through bubble bursting processes and survive aerosolization and exposure to cold temperatures, and that the microalga-biont particles could react to aerosolization conditions producing VOCs and actively nucleate ice at warm subzero temperatures. Thus, *L. gaiensis* is a potential candidate for air transportation and dispersal, with possible effects on atmospheric processes. We provide, for the first time, estimates of *L. gaiensis* emission fluxes.

Microalgal aerosolization has previously been reported. *Chlamydomonas*-like species were detected in air samples, snow depositions, and in artificial water tanks following air deposition. In this study, we showed that *L. gaiensis* could be aerosolized from water sources under bubble bursting using either a single or multiple jets. The emitted concentration of the microalgae ensemble was constant and reached up to 4 × 10^7^ cells.m^−3^. This emission rate is far higher than natural emission rate records on microalgae (≤3 × 10^3^ cells.m^−3^)^4^ and picomicroalgae (≤3.7 × 10^5^ cells.m^−3^)^12^.

Natural fluxes at lake scale are difficult to calculate as little is known on the typical limnic concentrations of *L. gaiensis* or *Chlamydomonas* sp. One of the reasons is that abundances in limnic surveys are often provided as dry weight or biomass^34,35^, qualitative measurements^35^, or binary data (presence/absence) with ecological information^36^. Emission rates were estimated in studies using either natural and mock phytoplankton community assemblages or culture enrichments loaded in tanks with microalgal concentrations up to ~10^3^−10^7^ cells.mL^−1^, at the upper ranges of natural phytoplankton blooms^10,11,37,38^. In our study, we used similar concentration ranges spanning ~10^3^−10^8^ cells.mL^−1^. In our cell counts, we noticed lower emission of microalgae (3.2%) in densely populated experiments (10^12^ cells, Exp5) than in less dense water sources ( ~ 10^7^ cells, 44.8% of EM, Exp1–4). The abundance contributes but does not fully explain the higher emission rate retrieved in *L. gaiensis*, suggesting that other intrinsic and extrinsic factors play a role in microalgal aerosolization (e.g., droplet characteristics, organism size, salinity, growth, and habitat conditions).

Particles aerosolization potential can be influenced by organismal properties^39^. Jet droplets can up-concentrate 8–307 times available aquatic microalgal density and this up-concentration is taxa-specific^11^. Organism size can play a role, as the highest emission estimates were reported in picophytoplankton^12^. Organism growth and the production of exudates can also increase the number of aerosols^10^. Exudates change the viscosity of the water surface and could affect the number of generated droplets^40^. Unlike many microalgal taxa (e.g., cyanobacteria, diatoms), *L. gaiensis* is not known for mucilage or exudates production^1^. Thus, a combination of organism size and exudate production from the microalgae entity could influence organismal density load per droplet, and thus their emission rate. This hypothesis calls for further investigations, as the load of *L. gaiensis* per droplet was not investigated, falling behind the scope of this study.

The concentration and size of generated aerosols can differ by the technique and the intensity of the stressor used to produce them^41,42^. In our study, we used bubble bursting, an aerosolization technique known to produce a broad size distribution and being able to efficiently eject microalgae up to 13 cm into the air^37^. We induced bubble bursting using single and multiple jets, and generated bubbles that covered the whole water surface area of the tank. Pietsch et al.^42^ showed a positive correlation between wind speed (water turbulence) and flux of aerosolized aquatic microorganisms. The systematic but marginally lower number of EM in SJ9 than in MJ3, suggests that the microalgal emission would be greater under conditions comparable to the multiple jets setup. While this difference was not significant, we observed it both when investigating the full microalgae ensemble (OPS data) and when following the cell abundance in the water tank. Higher emissions using SJ compared to MJ had previously been observed for the used tank when analyzing water containing synthetic sea salt^43^. The authors observed a steady increase in particle flux with increasing water velocity, which is not in agreement with the constant fluxes observed in this study when using freshwater microalgae (Supplementary Fig. 2).

Aerosolized microalgae are candidate propagules for air dispersal. Along their journey, they are subject to diverse stressors. Water turbulences generated during bubble-bursting experiments accounted for the loss of about one-fourth of the aquatic population. The homogenization step led to a net diminution of the mean cell biovolume and an increase of at least 18.5% in the number of dead cells. Larger cells were not observed during treatments in the aquatic samples or on filters after aerosolization. Instead, a high number of broken cells and debris were visible from filters. It is possible that larger organisms have been damaged by applied pressures both in the water and on filters, or removed from the water surface by water turbulences, towards the bottom of the water tank. The latter corroborates with simulations^44^ showing that, against expectations, larger particles would sediment faster under turbulent flow. Ross^45^ showed that particles sedimented in well-homogenized surface mixed layer (SML) tend to resuspend at the bottom of the SML where higher turbulences occur. Here we used a water tank with a small SML (~27.1–30.1 cm) and sampled the top 2 cm. However, organisms with larger and elongated shaped have a higher encounter rate, facilitating agglomeration and sedimentation^46^. Moreover, to avoid deleterious effects of strong turbulence, motile phytoplankton species tend to evade turbulent layers^47^. Therefore, it is possible that cells with larger biovolume would be flushed and trapped at the bottom of the SML, a natural exclusion facilitated by the microalgal tendency to attach to particles and surfaces^1^.

*Limnomonas gaiensis* was further negatively selected during aerosolization with ~97.6% of dead cells retrieved from the emitted fraction. A small fraction of EM (2.4%) maintained their viability, some of which revived in conditions favoring growth. Interestingly, the survival rate was higher when the organisms were collected in liquid phase (~10.8%) than on solid-dry phase (0%). Similarly, higher recovery rates of airborne microorganisms were found when using liquid impinger than filters^48^. On filters, desiccation and osmolysis could have drastically reduced survival chances. Impinger technique appeared to be less destructive with few to no broken cells and debris. This suggests that a low proportion of *L. gaiensis* can generate viable airborne propagules, and that the duration of their transportation, i.e., period of organism exposure to additional atmospheric stressors^49^, may play a key role in determining their capacity and distance of dispersal.

The airborne survival of *L. gaiensis* is affected by subzero temperatures, at times by freezing events. In a previous study^23^, we showed that airborne and aquatic microalgae, among which *Chlorophyceae*, can cope with temperatures down to −28 °C. *Limnomonas gaiensis* could tolerate gradual and shorter exposure down to −21 °C and experience freezing (this study). Despite negative selection, some *L. gaiensis* could survive thermal stress during air transportation and wet depositions.

Aerosolization survival to dispersal can be strain dependent. Airborne microalgae can adjust their physiological conditions to the environment, strain-specifically producing thicker cell wall and carotenoids to cope with UV exposure and reactive oxygen stress^15^. Strains of *L. gaiensis* were impacted by aerosolization treatments, especially when using multiple jets. Interestingly, there was a significant difference in the number of EM between strains. Also, the strain with the larger biovolume (VR66-07) had the highest emission rate when the microalgal load was ~10^7^ cells, whereas R86-47 had not only the smaller biovolume, but it also demonstrated a higher emission rate when the aquatic microalgal load was >10^9^ cells, and a better survival rate both after emission and freezing. Additionally, the negative trend between the percentage of revived organisms and the condition of microalgal growth (density, age) suggests that cell abundance and physiology may play an important role in the species survival capacity. The physiological response under aerosolization and freezing differed between strains, despite organismal concentration and growth phase. VR66-07 and R86-47 had similar revival capacities after cold exposure (*Z*-test *X*^2^_(1)_ = 7.45, *p* = 0.006) and their entity produce INA soluble proteins active below −17 °C, whereas R86-45 was less efficient at coping with cold temperature exposure (Z-test *X*^2^_(1)VR66-07- R86-45_ = 20.58 and *X*^2^_(1)R86-47- R86-45_ = 42.98, *p* < 0.001, respectively) and its entity produced non-soluble INA proteins active from −8 °C. To decipher the mechanisms behind *L. gaiensis* tolerance to atmospheric stressors, results call for complement morphological and physiological investigations.

Even though estimations suggest that air abundance and invasion risk of *L. gaiensis* are rather low, the microalga-biont natural complex could influence, to a certain extent, its environment producing VOCs and being IN active at warm subzero temperatures.

Emissions by aquatic organisms have been studied carefully, particularly regarding the production of dimethyl sulfide^50^, a molecule that plays a role in the formation of warm clouds (e.g., CLAW hypothesis^51,52^). Our study shows that *L. gaiensis* entity can produce low amounts of ethanol under stress induced by bubble bursting. Gas-phase ethanol reacts in the atmosphere with hydroxyl radicals to form acetaldehyde, thereby potentially contributing to ozone and smog formation. This observation is of great interest as the sources of ethanol in the atmosphere remain so-far poorly quantified.

Interestingly, we found higher concentrations of released ethanol using single than multiple jets. Ethanol is produced through the fermentation of microalgal polysaccharides such as glycogen, cellulose and starch, the latter being accumulated in green microalgae (as *Chlamydomonas* sp.) in 50% up to 70% of their dry weight^53,54^. Starch accumulation enhancement can be triggered under microelement limitation coupled with air bubbling^54^, diverting energy away from cell growth^55^. Ethanol can serve as a carbon source for other microbes and acts in the microalgae as antioxidant or as stimulant of metabolite accumulation and for the production of lipid, fatty acids, and biomass^56^. While ethanol can be beneficial for the microalgae, it can also alter cell metabolism, oxidative stress response, and growth. This effect is species-dependent and has been monitored in different taxa in the range of 0.39 to 16 g L^−1^. In our study, the concentration of ethanol released in the air was low (≤2 × 10^−4 ^g L^−1^), suggesting that ethanol would rather be a stimulating defense mechanism than an inhibitor of organismal activity.

In the atmosphere, cloud condensation and ice nucleation properties of PBAPs can favor the formation of clouds and wet deposition^28^, affecting the distance of air transportation of microorganisms (including microalgae) over geographic scales^3^. Even though they are a minority among aerosols, emitted marine PBAPs, of a size of 0.5–10 µm, can act as giant CCN, influencing precipitation^57^. Additionally, ice nucleation in microalgae has been reported in the past (see papers herein), including in *Chlamydomonas*-like species. The *L. gaiensis* entity could actively nucleate ice at temperatures ≤−18 °C, triggered by soluble INA proteins of a size <0.22 µm (in 3/4 strains). Their activity occurred at similar temperatures as in other aquatic microalgal species (−18 to −24 °C, e.g., *Peridinium aciculiferum* and *Microcystis* sp.) with higher efficiency, triggered by a lower number of IN particles ( ~ 10^−5^ INP.cell^−1^ in *L. gaiensis* versus ~10^−1^–10^−2^ INP.cell^−1^)^23^. However, this activity occurred at lower temperatures than reported in *Chlamydomonas* sp. (−8 and −17 °C)^25^. The low IN activity detected in R86-45 from −8 °C (2.1 × 10^-6^ INP.cell^−1^) was possibly trigged by cell-attached INA proteins.

As microalgae live in close relationship with their bionts^58^, we suspect that the microalgal entity, rather than the microalgal alone, is aerosolized. It is therefore possible that the cell-attached INA proteins were not of microalgal origin but were produced by the few prokaryotes attached to the microalgal cells or present in the phycosphere. It has been proposed that attached-bionts can be IN active for the entity^59^ or contribute to the microalga IN activity^25^. Nevertheless, microalgal axenic and non-axenic cultures can be active in the same temperature ranges^23^. It would be interesting to perform complement analyses on R86-45 to decipher the source of the activation.

The low activity observed in the *L. gaiensis* entity suggests that its contribution to atmospheric processes is negligeable in comparison to efficient PBAPs (active from −1 °C)^60^ and inorganic particles in general (<−15 °C)^28,61^ present in the atmosphere. However, the ice nucleation properties of *L. gaiensis* entity could confer advantages contributing to its survival and to reducing its residence time in the atmosphere.

The distribution of *Chlamydomonas* sp. covers a wide range of biogeographic regions around the world^3^, but very little is known on their atmospheric abundance. Microalgal airborne concentrations are usually very low, i.e., up to 10^4^ cells.m^−3^ over the North Atlantic Ocean^14^ and hundreds of cells.m^-3^ locally^9,62^, sometimes below detection limit^63^. Only a fraction of the emitted microorganisms can remain airborne (10% after 4 days after emission), allowing long distance travel up to 10^4 ^km of 0.5-5 µm unicellular eukaryotes^14^. Their emission and chance of long-distance transportation can be increased under conditions stimulating small droplet emission^42^. Additionally, environmental selection pressures, dilution factors during dissipation and transportation, and species selective requirements to freshwater habitats may negatively select the pool of viable aerosolized propagules. Generated data in the present study can serve as inputs in models to assess the potential transportation range of *L. gaiensis*. Altogether, the capacity of *L. gaiensis* to be aerosolized, to possibly produce ethanol and INA molecules, to cope with cold temperatures, freezing events, and microbial exposure even to toxic species, to carry bionts (reported here and from the literature), and its potential for spreading and acclimation^1,2^, makes this species a non-negligeable actor in the environment and the society^9,16^. Available knowledge calls for further investigations on *L. gaiensis* survival capacities to atmospheric stressors, its biont diversity, and an evaluation of the viable distance of its propagule dispersal.

## Methods

### Strains

**Fig. 7:**
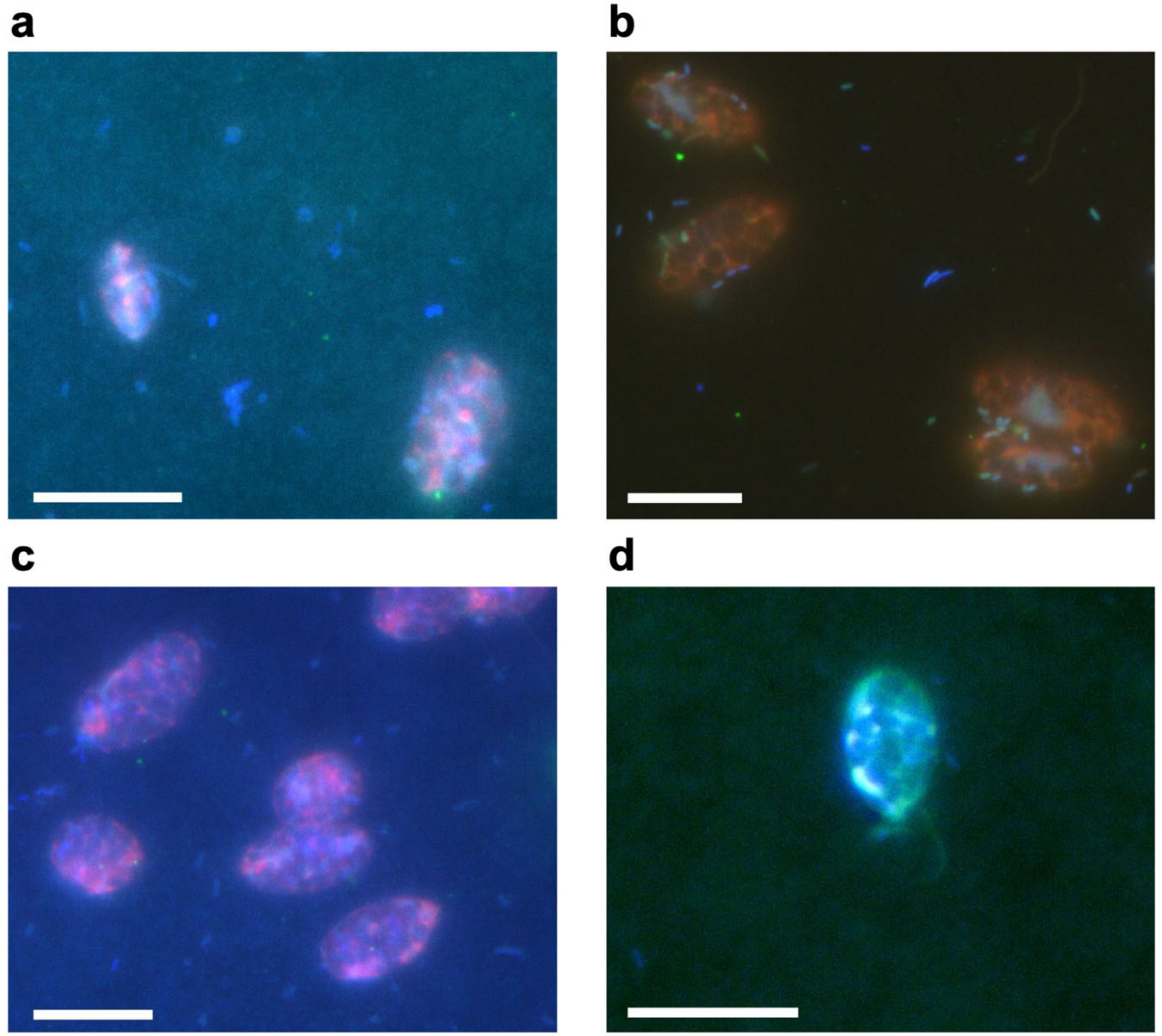
*Limnomonas gaiensis* and prokaryotic bionts in cultures. **a**: VR66-07, **b**: R86-47, **c**: VR66-10, and **d**: R86-45. Scale bar: 10 µm. Microalgal cells appeared red because of their chlorophyll autofluorescence, and their DNA in blue stained by DAPI. Prokaryotes, smaller in size, appeared blue by DNA staining and green in **b** (universal probe).

Strains of the freshwater microalga *Limnomonas gaiensis* (VR66-07 and R86-47) originating from forest lakes in Sweden^1^ were grown in MWC medium^64^ in a controlled climate chamber at 15 °C, with a 12 h dark:12 h light regime and 50 µmol photons.m^-2^.s^-1^ light intensity. Strains were non-axenic (Fig. 7), mimicking the species natural entity (i.e., microalga and bionts). Prokaryotes were present in the medium of culture, dispatched as single cells or as groups of cells; few were attached to *L. gaiensis*; and others were present in its phycosphere. Two additional non-axenic strains (VR66−10 and R86-45), originated from the same forest lakes in Sweden^1^, were used for investigating the effect of exposure to warm subzero temperatures (see below). The four strains are available at the Culture Collection of Algae at the University of Göttingen, Germany (SAG 2636−2639).

### Aerosolization settings

A stainless-steel tank was used to investigate the strain aerosolization simulating natural bubble bursting, as described in ref. ^65^. The tank was filled with 10 L of MilliQ water (EMD Millipore, 18.2 MΩ.cm at 25 °C, 2 ppb TOC). Bubbles were generated either by a single centered 4 mm plunging jet (SJ) or by multiple jets (MJ) composed of eight nozzles of 2 mm each. Water flow rates were set to 6.0 and 6.2 L min^−1^ (SJ7 and SJ9, respectively, named after the pump setting) in the SJ treatment and 8.7 and 11.4 L min^−1^ (MJ3 and MJ5, respectively) in the MJ treatment. Flow velocities translated to 8 m.s^−1^ under SJ- and 6–7 m.s^−1^ under MJ treatment^43^. A concentration of 10^6^–10^11^ microalgal cells.L^−1^ was loaded in the tank (Table 1), mimicking microalgal concentrations naturally occurring in lake during a bloom of *Chlamydomonas* sp.^34^ and previously used in aerosolization experiments^38^. Emitted gases and particles were sampled from the headspace (ca. 5 L), above the water column.

### Emission fluxes

Continuous aerosolized concentrations of particles in the size range 0.30–10 µm were measured using an Optical Particle Spectrometer (OPS 3330, TSI, flow rate: 1 L.min^−1^). A silica gel diffusion dryer was placed in front of the instrument to assess the concentration of dry particles. The total concentration of particles was then used to calculate the emission flux of the “microalgae ensemble” (i.e., cells, cell fragments and other microalgal associated material). The flux (F) was estimated using Eq. 1, taking into the account the sweep air through the system (Q_air_), total concentration of particles (N_OPS_), and the tank surface area (*A*_tank_ of 3.6 × 10^−2^ m^2^)^43^. Potential wall and bottom effects were not considered. Data was analyzed using AIM Software 10 (TSI).

1 $$F=\frac{{Q}_{{air}}{N}_{{OPS}}}{{A}_{{{{{{\rm{tan }}}}}}k}}$$

### Volatile organic compounds

Over the duration of each experiment (see Table 1), continuous measurements of the VOCs were taken in the m/z range 21–200 using a Proton Transfer Reaction Time-of-Flight mass spectrometer (PTR-ToF-MS 4000, Ionicon Analytik), operated under standard conditions with an E/N ratio of 132 Td with a drift tube volage of 820 or 900 V, a drift tube pressure of 3.3 or 3.4 mbar and a drift tube temperature of 80 or 60 °C. The PTR-ToF-MS was directly connected to the tank analyzing VOCs in the air above the water (headspace) with a resolution of 1 Hz. Data were analyzed with PTR-MS Viewer 3.2.12 (Ionicon).

### Microalgal abundance in the water tank

Microalgal abundance in the tank was evaluated over time and treatment to account for organismal airborne emission. A volume of 1 mL of aqueous sample was collected in triplicate from the tank after each treatment phase (i.e., starters, homogenized tank, SJ, MJ), fixed in neutral Lugol solution (4% final concentration, Bie & Berntsen A/S, Denmark), homogenized, and stored at 4 °C, in the dark, until further analyses. Cell abundance was assessed using a Sedgewick rafter counting chamber (S52, Graticules Optics Limited, United Kingdom) and an inverted microscope (AXIOVERT 135 M, Zeiss), magnification 100 or 200x.

### Revival capacities after aerosolization

Emitted organisms were recaptured for a period of 52-61 min on filters (PTFE hydrophilic membranes, 47 mm diameter, 0.20 µm pore size, H020A047A, Advantec) connected to a pump with a flow rate of ~2 L.min^−1^ or in 50 mL of sterile MWC using an impinger (NS 29/32 25×250 mm, Assistant, Duran), following Grinshpun et al.^66^ recommendations. After the collection period, filters were subdivided, under sterile conditions. Half of the filter was resuspended in 1 mL sterile MWC medium and fixed in 4% Lugol solution to assess cell abundance. A fourth of the filter was resuspended in 0.5 mL sterile MWC and dispatched in replicates in 96-well sterile microplates (Sarstedt, Germany) filled with 250 µL of sterile MWC, and incubated at 15 °C under light conditions favoring growth (as mentioned above) up to a period of two months. The last fourth of the filter was stored at 4 °C in the dark. For the impinger, a volume of 2 mL of homogenized liquid phase was fixed in duplicates in 4% Lugol solution and stored at 4 °C in the dark until cell abundance assessment, and a volume of 250 µL up to 500 µL was dispatched in replicates in either 96- or 48-well sterile microplates (Sarstedt and Cellstart® Greiner Bio-one) respectively. Inoculated plates were closed with parafilm to prevent wells from drying out and were incubated in a controlled culture chamber at 15 °C, 12 h dark:12 h light regime and 50 µmol photons.m^−2^.s^−1^ light intensity. To assess organismal revival through time, inoculates were monitored up to 2 months of incubation using an inverted microscope. Organismal revival and growth were regularly and qualitatively monitored for cell coloration, cell movement (swimming or pendulum movement^1^) and density using an inverted microscope (Axiovert 135 M, Zeiss).

### Microalgal biovolume

Microalgal biovolume (B, µm^3^) was determined following Eq. 2^67^ for prolate spheroid organisms, considering the organism width (W, in µm) and length (L, in µm).

2 $$B=\,\frac{\pi \,{W}^{2}\,L}{6}$$

### Cell vitality

Microalgal viability was tested using Neutral Red vital staining for microscopy (72210 Sigma-Aldrich) using 0.2–20% final concentration and 1:50,000 solution, following Zetsche and Meysman^33^ recommendations and Tesson and Šantl-Temkiv^23^ protocol. The dye accumulates in the cytoplasm and/or vacuoles of live organisms^68^ and provides an orange-red coloration to viable organisms.

Additionally, the concentration of total microalgae and of dead microalgae over treatments were estimated from a volume of 200 µL of homogenized culture and analyzed by flowcytometry using a NovoCyte 3000 flow cytometer equipped with a 405 nm, 488 nm, and 640 nm laser (Aligent, Santa Clara, CA). Note that performance and stability of the NovoCyte lasers and detectors are daily checked using NovoCyte QC Particles (Agilent). In each sample, a volume of 1.5 µL of propidium iodide (PI; 1 mg.mL^−1^, Sigma) was used to specifically stain dead cells. All samples were vortex shortly and loaded along negative controls (medium and autoclaved distilled water) on a 24-tube loader. Analysis was performed using a flow rate of 14 µL.min^−1^, a sample time of 1.26 min per sample, a 20 µL volume load, a series of 3 rinses between each sample, a homogenization of 1 cycle per sample with a speed of 1000 rpm for 10 sec, a forward scatter threshold for height set >50,000, and excitement with two lasers at 405 nm and 488 nm. Because the signal from both lasers was similar, we here show data from the 488 nm laser for comparison with available body of literature. Generated data was analyzed using FlowJo^TM^ version 10.8.1 (Becton Dickinson & Company 2006-2021).

### Ice nucleation activity, frozen fraction, and revival capacities after cold exposure and freezing events

Ice nucleation activity and freezing tolerance in *L. gaiensis* were investigated using a droplet-freezing assay^69^ in VR66-07 and R86-47, and in VR66-10 and R86-45, two strains collected from the same lakes^1^. Prior to the analyses, strains were grown at 4 °C, 50 µmol photons.m^−2^.s^−1^, 12 h dark: 12 h light. Twenty to thirty-two replicates of 20 µL of each of the four strains were loaded into 384-well plates (VWR International, Lutterworth, United Kingdom). Each plate also contained 20 to 32 replicates of INA *Pseudomonas syringae* R10.79 cells^70^ suspended in MWC (positive control) and 20–32 replicates of MWC (negative control). We used heat and filter treatments following the protocol described in ref. ^23^ to assess the nature of the INA compounds. A subset of cultures was filtered on polycarbonate membrane (Q-max syringe, Frisenette) to isolate particles of a size inferior to 0.22 µm (i.e., soluble fraction). A denaturation step by heat shock, of 10 min incubation at 100 °C, was used to reveal if the soluble particles and the total INA particles (soluble and particulate) had a proteinaceous origin^71^. Twenty to thirty-two replicates of 20 µL of each treatment were loaded alongside with the controls. The freezing profiles were determined using two biological replicates for a total of 52-64 replicates per treatment and per strain (*n* = two experiments × 20–32 replicates) and of 116 replicates for each control (*n* = two experiments × duplicate × 20–32 replicates), for each of the investigated temperature. Plates were incubated for 30 min in an environmental climate chamber (Binder MK115, Tuttingen, Germany) through a gradient of −4 to −21 °C, with an increment of −1 °C. The initial microalgal concentration span from 0.2 to 13.5 × 10^5^ cells.mL^−1^ (340–26,933 cells per inoculate). The fraction of frozen wells per condition and strains was monitored visually across the temperature gradient.

The frozen fraction (FF, i.e., proportion of frozen replicates for a given treatment and strain) was calculated using the equation from ref. ^72^. The FF of the negative control was subtracted to the FF of each sample. The number of ice nucleating particles (INP) per replicate was calculated using a modified equation from Vali^72^, described in^23^, taking into the account the concentration of microalgae per replicate. Negative values and measurements with less than three consecutive occurrences above the detection limit were removed from the FF and INP datasets. INP data provided in Fig. 6 were normalized to the negative controls. Graphical analyses were performed using the R software version 4.2.0^73^ and the packages ggplot2^74^, scales version 1.2.0^75^, dplyr^76^, and grid^77^. Investigated plates were then incubated at 4 °C under conditions favoring growth. Growth in each inoculates was monitored as mentioned above over a period of 23 days of incubation.

### Bionts

The presence of microalgal epibionts was investigated using Fluorescent In Situ Hybridization and fluorescent counter-staining. Cells were fixed by mixing one part of cellular growth with three parts of cold 4% PFA and incubating for 12 h or 24 h at 4 °C. Fixed samples were centrifugated at 14,000×*g* for 5 min at 4 °C and the pellet was washed in 1x phosphate-buffer saline (PBS) three times. Cells were resuspended in a 1:1 PBS:96% ethanol mixture and stored at -20 °C. Prior to cell hybridization, cells were filtered on a polycarbonate membrane filter (0.2 μm pore size). Cell hybridization was performed using EUB-MIX probe^78,79^ (0.5 ng DNA.µL^-1^) and the negative control NON-EUB probe^80^, both labeled with the fluorescent dye Atto-488 in 35% formamide for 2 h at 46 °C. Cells were then counterstained using 4′,6-diamidino-2-phenylindole (DAPI, 1 µg.mL^−1^), and mounted in Citifluor:Vecta Shield (4:1 v:v) medium. Microscopic imaging was performed using a fluorescence microscope at 400x magnification (Eclipse Ni, Nikon, Axiovert 200 M, Zeiss) and analyzed using the imaging software NIS-Elements (v.4.50, Nikon Instruments Inc., Melville, NY, USA).

#### Statistics and reproducibility

Statistical analyses were performed in R^73^. Comparison of means was performed using one-way and two-way ANOVA upon the assumption of homogeneous variances, tested using the F- and Levene tests, and a normal distribution of residuals, tested using the Shapiro-Wilk normality test. The post-hoc range test Tukey’s honestly significant difference (HSD) was used for multiple pairwise comparison with a confidence level of 95%. Upon inequality of variances, the non-parametric Kruskal–Wallis rank-sum test was used to determine if differences between groups were statistically significant, with a significant level alpha of 5%. Post hoc multiple pairwise comparisons were then performed using Dunn test and the R package FSA version 0.9.3^81^. Comparison of proportions was performed using *Z*-test with continuity correction for small sample size.

#### Inclusion and ethics statement

The research included local researchers throughout the research process; its relevance has been discussed among co-authors and with colleagues. Roles, responsibilities, and research plan were agreed amongst collaborators ahead of the research. The research was not severely restricted nor prohibited in the setting of the researchers. The research was based on microalgae cultures and was not approved by local ethics as it does not involve work on humans, animals, or dangerous substances. The research was undertaken to the higher standards and in accordance with Aarhus University and Danish regulations. The research did not result in stigmatization, incrimination, discrimination, or otherwise personal risk to participants. The research did not involve major health, safety, security or other risk to researchers, and basic laboratory safety measures were performed according to regulations at Aarhus University. No biological materials, cultural artefacts or associated traditional knowledge were produced in this research. Local and regional research relevant to our study were considered in citations.

### Reporting summary

Further information on research design is available in the Nature Portfolio Reporting Summary linked to this article.

### Supplementary information

Supplementary Information

Description of Additional Supplementary Files

Supplementary Data

Reporting Summary

## Data Availability

The datasets generated during and/or analyzed during the current study are available in the Supplementary data file and from the corresponding authors on reasonable request. Investigated strains are available at the Culture Collection of Algae at the University of Göttingen, Germany (SAG 2636-2639).
