# Author Correction: Aerosolization flux, bio-products, and dispersal capacities in the freshwater microalga *Limnomonas gaiensis* (Chlorophyceae)

**DOI:** 10.1038/s42003-023-05228-9

**Published:** 2023-08-14

**Authors:** Sylvie V. M. Tesson, Marta Barbato, Bernadette Rosati

**Affiliations:** 1 Aarhus Institute of Advanced Studies, Aarhus University, Aarhus, Denmark; 2 Department of Biology, Aarhus University, Aarhus, Denmark; 3 Department of Chemistry, Aarhus University, Aarhus, Denmark

**Keywords:** Ecology, Microbiology

Correction to: *Communications Biology* 10.1038/s42003-023-05183-5, published online 03 August 2023

In the original version of the article (in the Aerosolization section on page 3 and in the sentence beginning ‘The emission fluxes…’ the power sign of +4 is displayed incorrectly as –4 at 5 places.

The correct version of the number is 0.8–7.9 × 10^4^ m^–2^.s^–^^1^ which replaces the previous incorrect version: 0.8–7.9 × 10^−4^ m^−2^.s^−1^.

In the same line, the correct version of equation is (2.4 × 10^4^ m^−2^.s^−1^ ± 1.3 × 10^4^) which replaces the previous incorrect version: (2.4 × 10^−4^ m^−2^.s^−1^ ± 1.3 × 10^−4^).

Further down the same line, the correct version of equation is (4.8 × 10^4^ m^−2^.s^−1^ ± 2.4 × 10^4^) which replaces the previous incorrect version: (4.8 × 10^−4^ m^−2^.s^−1^ ± 2.4 × 10^−4^).

This has been corrected in the PDF version.

