# Supplementary material for: Aerosolization flux, bio-products, and dispersal capacities in the freshwater microalga Limnomonas gaiensis (Chlorophyceae)
Source: Commun Biol. 2023 Aug 3;6:809. doi: 10.1038/s42003-023-05183-5 (PMC10400582; doi:10.1038/s42003-023-05183-5)
Supplement: Supplementary file 1 — Supplementary Information [file 42003_2023_5183_MOESM1_ESM.pdf]

Supplementary Material

Aerosolization flux, bio-products, and dispersal capacities in the freshwater microalga *Limnomonas gaiensis* (Chlorophyceae).

Sylvie V.M. Tesson [1,2,*], Marta Barbato [2], Bernadette Rosati [3,*]

Affiliations:

1 Aarhus Institute of Advanced Studies, Aarhus University, Aarhus, Denmark

2 Department of Biology, Aarhus University, Aarhus, Denmark

3 Department of Chemistry, Aarhus University, Aarhus, Denmark

Corresponding authors *

Content:

1. Schema of the experimental setting (Supplementary Figure 1)
2. Pilot study: OPS results (Supplementary Figure 2)
3. Abundance of microalgal cells in the water tank (Supplementary Figure 3)
4. Emission flux as a function of cell abundance (Supplementary Figure 4)
5. *Limnomonas gaiensis’* revival capacity after exposure to subzero temperatures (Supplementary Figure 5)
6. Total cell and dead cell abundances in *Limnomonas gaiensis* in Experiment 7. (Supplementary Table 1)
7. Survival capacity in *Limnomonas gaiensis* after exposure to subzero temperatures and incubation at 4°C up to three weeks in condition favoring growth (Supplementary Table 2)

Supplementary Figure 1. Schema of experimental setting.

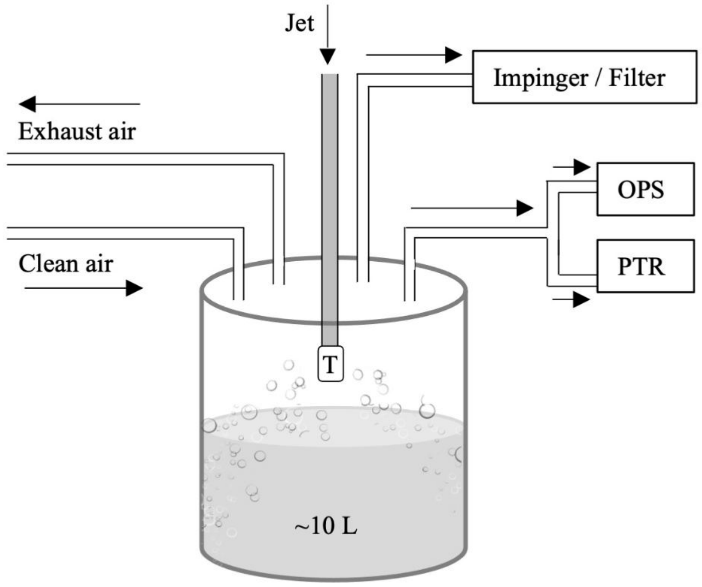

Water tank[1] containing approximately 10 L of MilliQ water and microalgae. Bubbles were produced by water jet impingement on the water surface. Either a single jet (SJ) or 8 multiple jets (MJ) were used for the treatments, located at the place marked with T in the setup. The headspace was continuously flushed with clean air. Aerosols were continuously monitored with an optical particle spectrometer (OPS) and either collected on a filter or an impinger. VOCs were measured with a proton transfer reaction time of flight mass spectrometer (PTR). An exhaust line ensured that no overpressure built up in the system.

Supplementary Figure 2. Assessment of water flow rate for total number concentration of aerosolized microalgae ensemble.

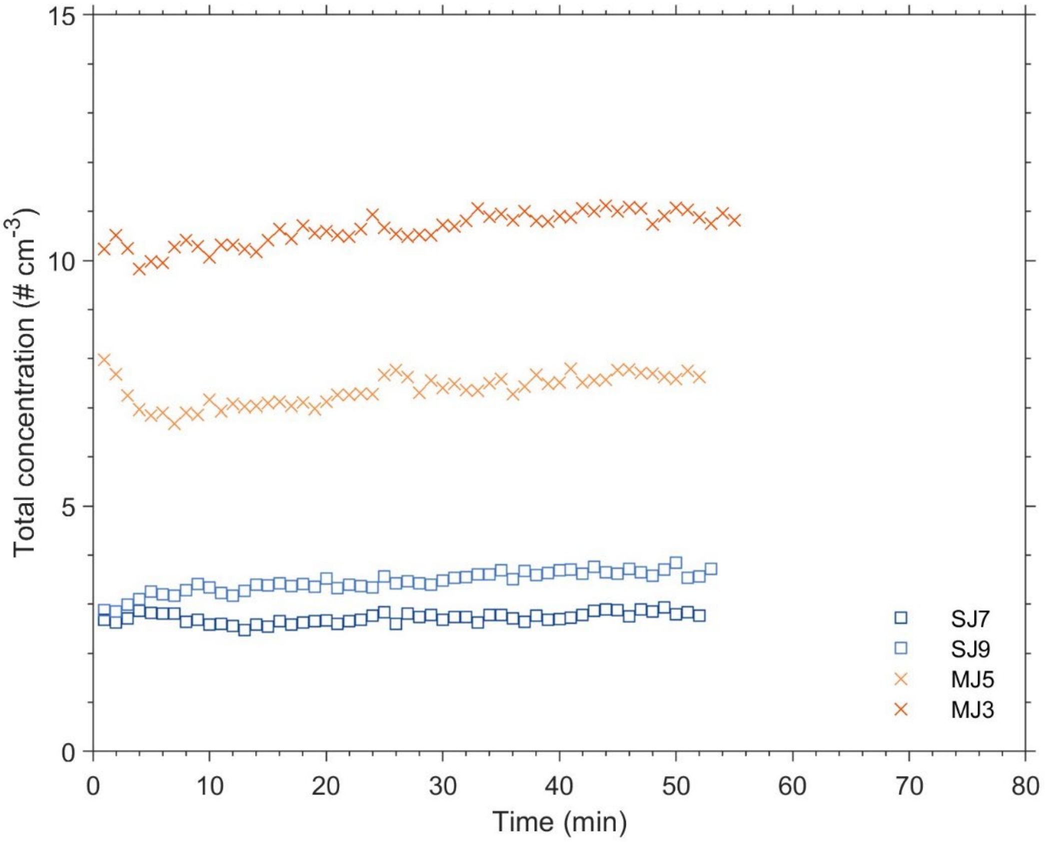

Bubble bursting were created using either a single jet treatment with a flow intensity of 7 (dark blue squares, n= 52) or 9 (light blue squares, n= 53) or a multiple jet treatment with a flow intensity of 5 (orange crosses, n= 52) or 3 (red crosses, n= 55) in the *Limnomonas gaiensis* strain R86-47 over Experiment 2. The pump of the bubble bursting simulation chamber was varied to assess good settings to carry out the following experiments.

Supplementary Figure 3. Abundance of microalgal cells in the water tank across treatment in two strains of *Limnomonas gaiensis* for the four flow intensities investigated.

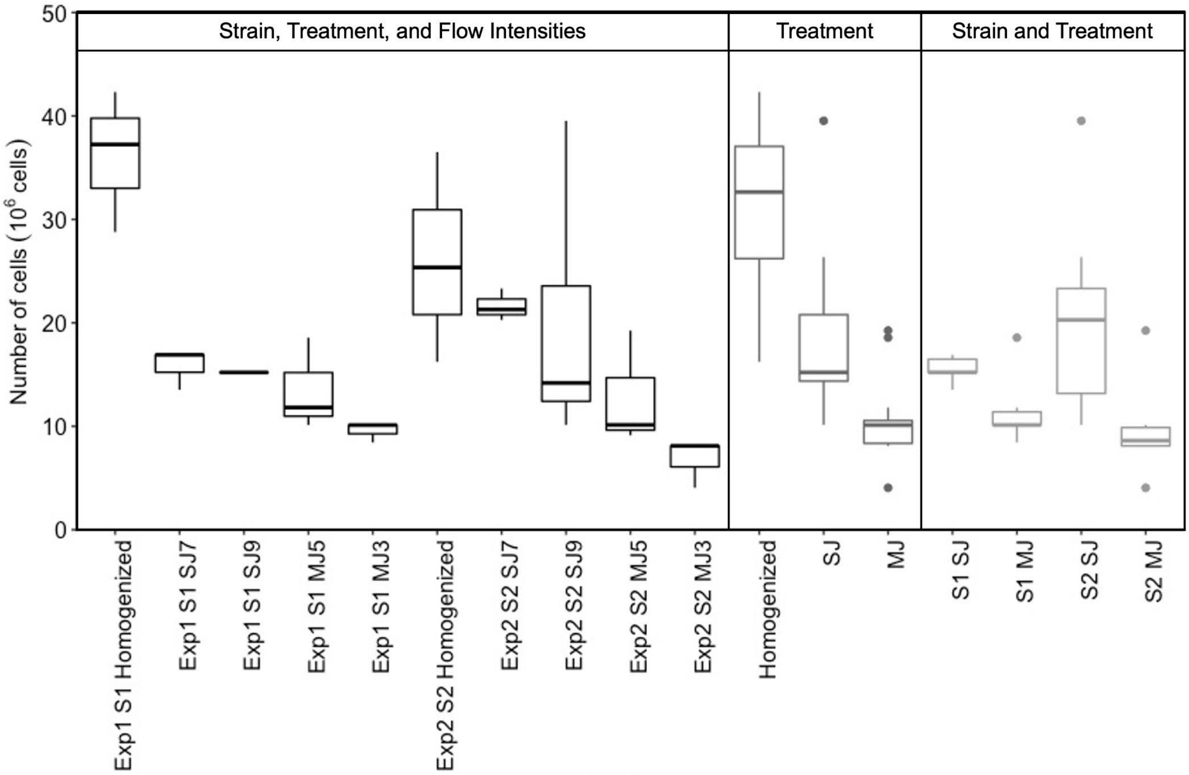

Flow intensities using either single (SJ) and multiple (MJ) jet treatments: SJ7, SJ9, MJ5, MJ3. Results from Exp1 for strain VR66-07 (denoted as S1) and from Exp2 for strain R86-47 (denoted as S2), in triplicates per condition. Technical duplicates were counted for Exp2 S2 SJ9. Grey: synthesis per treatment (n=6, 15, 12 replicates, respectively). Light grey: synthesis per treatment and strain (n=6, 9, 6, 6 replicates, respectively). Standard deviation denotes the variability between replicates within experiments (black bar), between treatments (grey bars) and treatment and strain (light grey bars).

Supplementary Figure 4. Emission fluxes in *Limnomonas gaiensis* displayed as a function of initial cell abundance.

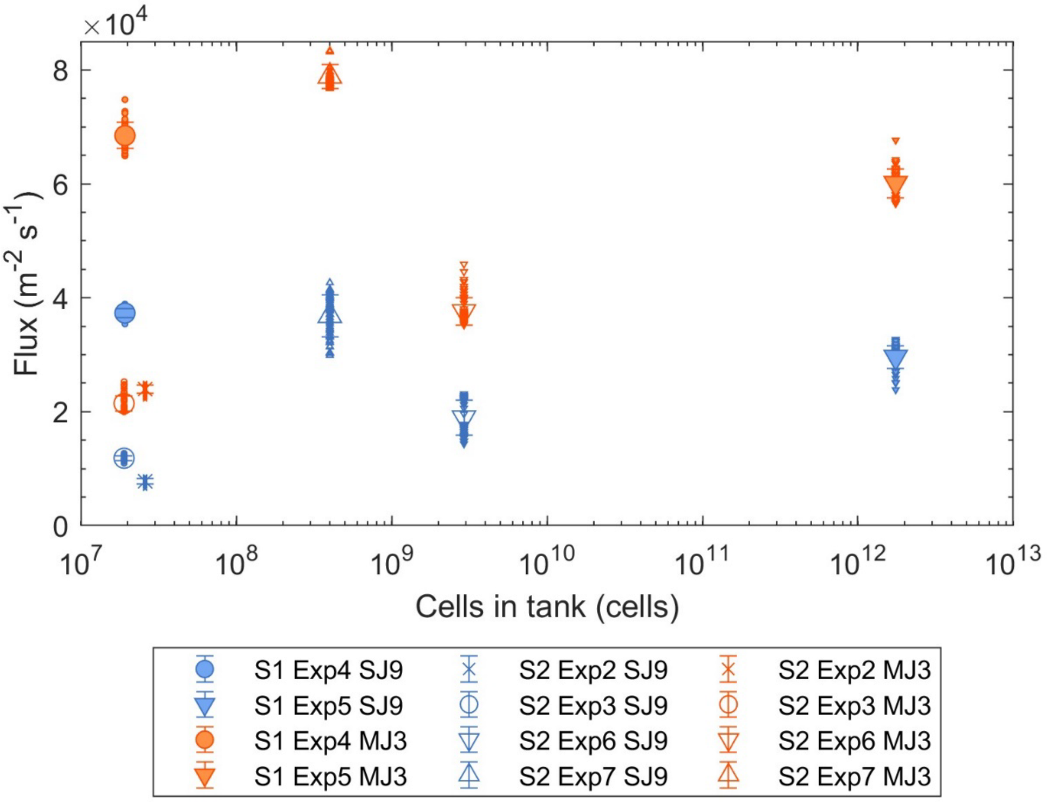

Strains: VR66-07 denoted as S1 (plain symbol) and R86-47 as S2 (empty symbol). Treatments: SJ9 (blue) and MJ3 (orange). The shape of the symbol show results for Experiment 2 (cross), Experiments 3-4 (circle), Experiments 5-6 (upside-down triangle), Experiment 7 (upside-up triangle). The number of observations per strain and treatment ranges from 52-58.

Supplementary Figure 5. Revival capacity in strains of *Limnomonas gaiensis* after exposure to subzero temperatures as a function of the inoculate size.

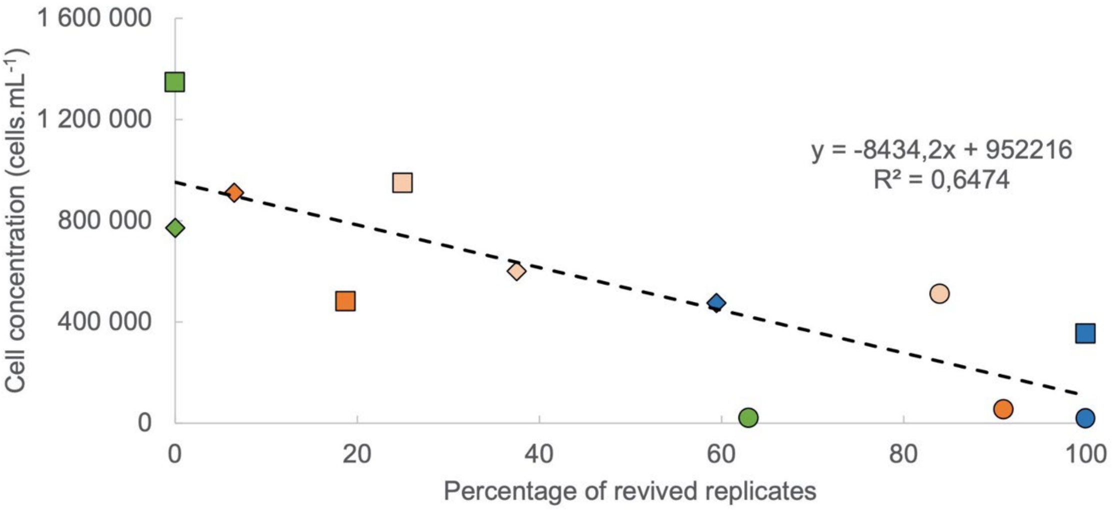

Strains: R86-45 (green), R86-47 (blue), VR66-07 (light orange) and VR66-10 (dark orange). Culture status: aged (squares), refreshed-aged (diamonds), and young (circles). Trendline: linear regression. Thirty-two replicates per strain and culture status.

Supplementary Table 1. Total cell and dead cell abundances in *Limnomonas gaiensis* in Experiment 7.

Samples	Treatment	Total cell abundance	Average	Abundance of dead cells	Average	Percentage of dead cells
ST1	initial culture	978692550		65995050		
ST2	initial culture	1130492550		74571750		
ST3	initial culture	917479200	1008.9 10^6 ± 109.7	68993100	69.9 10^6 ± 4.4	7.0 ± 0.5
A	Homogenization	1605124300		382198050		
B	Homogenization	1684144150		390261300		
C	Homogenization	1065424100	1451.6 10^6 ± 336.7	313929200	362.1 10^6 ± 41.9	25.5 ± 3.5
D	SJ	3038714250		902210650		
E	SJ	2654509000		683509200		
F	SJ	2418075000	2703.8 10^6 ± 313.2	716287550	767.3 10^6 ± 118.0	28.4 ± 2.3
G	MJ	2441346750		684866250		
H	MJ	2403209100		706889400		
I	MJ	2582617200	2475.7 10^6 ± 94.5	678420450	690.1 10^6 ± 14.9	27.9 ± 1.6
IMPA1	SJ	*0*		*0*		
IMPA2	SJ	*5000*		*5000*		
IMPA3	SJ	*7500*	*4166.7 ± 3818.8*	*7500*	*4166.7 ± 3818.8*	*100.0 ± 0.0*
IMPB1	MJ	*5000*		*5000*		
IMPB2	MJ	20000		20000		
IMPB3	MJ	17500	14166.7 ± 8036.4	15000	13333.3 ± 7637.6	*95.2 ± 8.2*<br>92.9 ± 10.1

The Total cell abundance in each replicate (in cells) and the respective average and standard deviation for each treatment (triplicates); the Abundance of dead cells in each replicate (in cells) and the respective average and standard deviation for each treatment (triplicates); and the average and standard deviation of the resulting Percentage of dead cells (in %). Samples below threshold of detection are indicated in italic. The percentage of dead cells for IMPB in italic takes into the account all IMPB replicates above and below the detection threshold.

Supplementary Table 2. Tolerance capacity in *Limnomonas gaiensis* to subzero temperatures and after incubation at 4°C for up to three weeks in condition favoring growth.

	Aged culture			Refreshed Aged culture			Younger culture		
Strains	N	C	R	N	C	R	N	C	R
R86-45	2.69 ± 0.82	134.7	0.0	1.55 ± 0.23	77.3	0.0	0.041 ± 0.0009	2.0	62.5
R86-47	0.71 ± 0.02	35.4	100.0	0.95 ± 0.34	47.6	59.4	0.034 ± 0.003	1.7	100.0
VR66-07	1.90 ± 0.76	95.0	25.0	1.20 ± 0.36	60.1	34.4	1.02 ± 0.42	50.9	84.4
VR66-10	0.96 ± 0.37	48.1	18.8	1.82 ± 0.18	91.1	6.5	0.11 ± 0.025	5.3	90.6

The cell abundance in each replicate (N, 10^4 cells), cell concentration (C, 10^4 cells.mL^{-1}) and percentage of revived replicates per strain (R, %).

Supplementary References

[1] King, S.M. et al. Investigating Primary Marine Aerosol Properties: CCN Activity of Sea Salt and Mixed Inorganic–Organic Particles. *Environmental Science & Technology* **46**, 19, 10405-10412 (2012). https://doi.org/10.1021/es300574u
