# Supplementary material for: Aerosolization flux, bio-products, and dispersal capacities in the freshwater microalga Limnomonas gaiensis (Chlorophyceae)
Source: Commun Biol. 2023 Aug 3;6:809. doi: 10.1038/s42003-023-05183-5 (PMC10400582; doi:10.1038/s42003-023-05183-5)
Supplement: Supplementary file 2 — Description of Additional Supplementary Files [file 42003_2023_5183_MOESM2_ESM.pdf]

Description of Additional Supplementary Files

File name: Supplementary Data

Description: Processed data behind the main Figures 1,2,4,5, and 6 in the paper and Supplementary Figures 2, 3, 4, and 5 in the Supplementary material.
