# Supplementary material for: Aerosolization flux, bio-products, and dispersal capacities in the freshwater microalga Limnomonas gaiensis (Chlorophyceae)
Source: Commun Biol. 2023 Aug 3;6:809. doi: 10.1038/s42003-023-05183-5 (PMC10400582; doi:10.1038/s42003-023-05183-5)
Supplement: Supplementary file 4 — Reporting Summary [file 42003_2023_5183_MOESM4_ESM.pdf]

nature portfolio

Corresponding author(s): Sylvie Tesson

Last updated by author(s): Jul 20, 2023

Reporting Summary

Nature Portfolio wishes to improve the reproducibility of the work that we publish. This form provides structure for consistency and transparency in reporting. For further information on Nature Portfolio policies, see our Editorial Policies and the Editorial Policy Checklist.

Statistics

For all statistical analyses, confirm that the following items are present in the figure legend, table legend, main text, or Methods section.

n/a	Confirmed	
☐	☒	The exact sample size (*n*) for each experimental group/condition, given as a discrete number and unit of measurement
☐	☒	A statement on whether measurements were taken from distinct samples or whether the same sample was measured repeatedly
☐	☒	The statistical test(s) used AND whether they are one- or two-sided<br>*Only common tests should be described solely by name; describe more complex techniques in the Methods section.*
☐	☒	A description of all covariates tested
☐	☒	A description of any assumptions or corrections, such as tests of normality and adjustment for multiple comparisons
☐	☒	A full description of the statistical parameters including central tendency (e.g. means) or other basic estimates (e.g. regression coefficient) AND variation (e.g. standard deviation) or associated estimates of uncertainty (e.g. confidence intervals)
☐	☒	For null hypothesis testing, the test statistic (e.g. *F*, *t*, *r*) with confidence intervals, effect sizes, degrees of freedom and *P* value noted<br>*Give P values as exact values whenever suitable.*
☒	☐	For Bayesian analysis, information on the choice of priors and Markov chain Monte Carlo settings
☒	☐	For hierarchical and complex designs, identification of the appropriate level for tests and full reporting of outcomes
☒	☐	Estimates of effect sizes (e.g. Cohen's *d*, Pearson's *r*), indicating how they were calculated

Our web collection on statistics for biologists contains articles on many of the points above.

Software and code

Policy information about availability of computer code

Data collection	not applicable
Data analysis	AIM Software 10 (TSI) to estimate emission fluxes; PTR-MS Viewer 3.2.12 (Ionicon) to analyze volatile organic compound profiles; FlowJoTM 555 version 10.8.1 (Becton Dickinson & Company 2006-2021) to analyze flow cytometry data; R software version 4.2.0 for data visualization and statistical analyses; NIS-Elements (v.4.50, Nikon Instruments Inc.,<br>599 Melville, NY, USA) to visualize hydridized cells and microorganisms interactions under microscope.

For manuscripts utilizing custom algorithms or software that are central to the research but not yet described in published literature, software must be made available to editors and reviewers. We strongly encourage code deposition in a community repository (e.g. GitHub). See the Nature Portfolio guidelines for submitting code & software for further information.

Data

Policy information about availability of data

All manuscripts must include a data availability statement. This statement should provide the following information, where applicable:

- Accession codes, unique identifiers, or web links for publicly available datasets
- A description of any restrictions on data availability
- For clinical datasets or third party data, please ensure that the statement adheres to our policy

We wrote "The datasets generated during and/or analyzed during the current study are available in the Supplementary data file and from the corresponding authors on reasonable request."

Research involving human participants, their data, or biological material

Policy information about studies with human participants or human data. See also policy information about sex, gender (identity/presentation), and sexual orientation and race, ethnicity and racism.

Reporting on sex and gender	not applicable
Reporting on race, ethnicity, or other socially relevant groupings	not applicable
Population characteristics	not applicable
Recruitment	not applicable
Ethics oversight	not applicable

Note that full information on the approval of the study protocol must also be provided in the manuscript.

Field-specific reporting

Please select the one below that is the best fit for your research. If you are not sure, read the appropriate sections before making your selection.

☐ Life sciences ☐ Behavioural & social sciences ☒ Ecological, evolutionary & environmental sciences

For a reference copy of the document with all sections, see nature.com/documents/nr-reporting-summary-flat.pdf

Ecological, evolutionary & environmental sciences study design

All studies must disclose on these points even when the disclosure is negative.

Study description	We investigated a newly described species of a freshwater green microalga (Limnomonas gaiensis) for which the dispersal mode is not known and its water dispersal is not probable. We hypothesized that the species could be airborne dispersed and investigated step-by-step its (i) aerosolization capacity (i.e., relative magnitudes of aerosolization fluxes) for different types of jets, (ii) effect on atmospheric chemistry (i.e., production of volatile organic compounds and of ice nucleation active compounds from -4 down to -21° C) that can influence atmospheric processes, and (iii) viable capacities after aerosolization and freezing events.
Research sample	Investigations were performed on two to four strains of Limnomonas gaiensis, originated from two freshwater lakes in Sweden. These strains are deposited in culture collection.
Sampling strategy	Strains are originated from two lakes in Sweden. To perform the aerosolization tests we used initial culture concentrations in the upper range of natural phytoplankton bloom occurrences.
Data collection	Tesson provided the initial microalgal cultures (culture maintenance and cell abundance estimates using flow cytometry and microscope counts using life/dead staining); performed the droplet freezing assays (environmental culture chamber) and investigated the nature of ice nucleation active compounds using heat and filtration treatments; collected the samples and cell abundance data (flow cytometry and microscope counts using life/dead staining) from the aerosolization experiments; and performed the survival investigations after cold and aerosolization treatments (incubation and microscope observations).<br>Rosati provided atmospheric variables and data outputs from the aerosolization experiments to estimate the total number of emitted particle concentrations and fluxes (Optical Particle Spectrometer; OPS) and chemical profiles (nature and concentrations) of produced volatile organic compounds (Proton Transfer Reaction Time-of-Flight mass spectrometer; PTR).<br>Barbato performed cell hybridization (DAPI and universal probes) to investigate the presence and location of the microalgal bionts.
Timing and spatial scale	The experiments were run successively between 2021 and 2022. Timing or spatial scale are not applicable.

Data exclusions | Data from the PTR and OPS in the aerosolization experiment 1 were excluded due to the occurrence of a malfunction in the apparatus. An explanation was provided in the manuscript.

Reproducibility | All attempts to repeat the experiments were successful.

Randomization | Strains were tested in parallel in the droplet freezing assays, survival tests, and life/dead staining, using biological and technical replicates. Due to technical limitations, strains were tested on different days in the aerosolization experiments (most often one day after the other). The strain order was randomly chosen.

Blinding | Collected samples were labeled according with the strain information, experimental date and treatments. During data acquisition all samples were treated similarly. Factors and variables were considered during data analyses.

Did the study involve field work? ☐ Yes ☒ No

Reporting for specific materials, systems and methods

We require information from authors about some types of materials, experimental systems and methods used in many studies. Here, indicate whether each material, system or method listed is relevant to your study. If you are not sure if a list item applies to your research, read the appropriate section before selecting a response.

Materials & experimental systems

n/a	Involved in the study
☒	☐ Antibodies
☒	☐ Eukaryotic cell lines
☒	☐ Palaeontology and archaeology
☒	☐ Animals and other organisms
☒	☐ Clinical data
☒	☐ Dual use research of concern
☒	☐ Plants

Methods

n/a	Involved in the study
☒	☐ ChIP-seq
☐	☒ Flow cytometry
☒	☐ MRI-based neuroimaging

Flow Cytometry

Plots

Confirm that:

☒ The axis labels state the marker and fluorochrome used (e.g. CD4-FITC).

☒ The axis scales are clearly visible. Include numbers along axes only for bottom left plot of group (a 'group' is an analysis of identical markers).

☒ All plots are contour plots with outliers or pseudocolor plots.

☒ A numerical value for number of cells or percentage (with statistics) is provided.

Methodology

Sample preparation | Samples of 200 µL of homogenized sample (from controls, cultures, aerosolized samples or water tank) were investigated using 1.5 µL of propidium iodide (1 mg/mL, Sigma) to specifically stain dead/damaged cells

Instrument | Novocyte 3000 (Aligent, Santa Clara, CA)

Software | FlowJoTM version 10.8.1 (Becton Dickinson & Company 2006-2021)

Cell population abundance | We studied microalgal strain. Microalgae were the only particles that could emit a chlorophyll signal. Signal was corrected from negative controls to remove background signals. Collected samples were check for sample quality. Samples with abundances below the detection threshold (negative controls; blanks) were not considered for further analyses. Minimal population abundances were these above the threshold of detection. Abundance of dead/damaged cells was assessed through treatments using propidium iodide among all chlorophyll-containing particles.

Gating strategy | Gating of samples were determined in preliminary tests with and without propidium iodide, using positive (with microalgae) and negative (water and culture medium) controls, using replicates, and further adjusted across treatments in all experiments. To visualized the data log scale was used. Positive signals for propidium iodide and chlorophyll were > 10 to the power 4 and < 10 to the power 7.

☐ Tick this box to confirm that a figure exemplifying the gating strategy is provided in the Supplementary Information.
